# Development of a homotrimeric PSMA radioligand based on the NOTI chelating platform

**DOI:** 10.1186/s41181-024-00314-7

**Published:** 2024-12-11

**Authors:** Sebastian Martin, Moritz-Valentin Schreck, Tobias Stemler, Stephan Maus, Florian Rosar, Caroline Burgard, Andrea Schaefer-Schuler, Samer Ezziddin, Mark D. Bartholomä

**Affiliations:** 1 Department of Nuclear Medicine and Molecular Imaging, Lausanne University Hospital, Rue de Bugnon 25A, 1011 Lausanne, Switzerland; 2 Department of Nuclear Medicine, Saarland University – Medical Center, Kirrbergerstrasse, 66421 Homburg, Germany; 3 Department of Nuclear Medicine, Saarland University, Kirrbergerstrasse, 66421 Homburg, Germany

**Keywords:** NOTI, Bifunctional chelator, Gallium-68, PSMA, Trimer, Multimerization

## Abstract

**Background:**

The NOTI chelating scaffold can readily be derivatized for bioconjugation without impacting its metal complexation/radiolabeling properties making it an attractive building block for the development of multimeric/-valent radiopharmaceuticals. The objective of the study was to further explore the potential of the NOTI chelating platform by preparing and characterizing homotrimeric PSMA radioconjugates in order to identify a suitable candidate for clinical translation.

**Results:**

Altogether, three PSMA conjugates based on the NOTI-TVA scaffold with different spacer entities between the chelating unit and the Glu-CO-Lys PSMA binding motif were readily prepared by solid phase-peptide chemistry. Cell experiments allowed the identification of the homotrimeric conjugate **9** comprising NaI-Amc spacer with high PSMA binding affinity (IC_50_ = 5.9 nM) and high PSMA-specific internalization (17.8 ± 2.5%) compared to the clinically used radiotracer [^68^Ga]Ga-PSMA-11 with a IC_50_ of 18.5 nM and 5.2 ± 0.2% cell internalization, respectively. All ^68^Ga-labeled trimeric conjugates showed high metabolic stability in vitro with [^68^Ga]Ga-**9** exhibiting high binding to human serum proteins (> 95%). Small-animal PET imaging revealed a specific tumor uptake of 16.0 ± 1.3% IA g^−1^ and a kidney uptake of 67.8 ± 8.4% IA g^−1^ for [^68^Ga]Ga-**9**. Clinical PET imaging allowed identification of all lesions detected by [^68^Ga]Ga-PSMA-11 together with a prolonged blood circulation as well as a significantly lower kidney and higher liver uptake of [^68^Ga]Ga-**9** compared to [^68^Ga]Ga-PSMA-11.

**Conclusions:**

Trimerization of the Glu-CO-Lys binding motif for conjugate **9** resulted in a ~ threefold higher binding affinity and cellular uptake as well as in an altered biodistribution profile compared to the control [^68^Ga]Ga-PSMA-11 due to its intrinsic high binding to serum proteins. To fully elucidate its biodistribution, future studies in combination with long-lived radionuclides, such as ^64^Cu, are warranted. Its prolonged biological half-life and favorable tumor-to-kidney ratio make this homotrimeric conjugate also a potential candidate for future radiotherapeutic applications in combination with therapeutic radionuclides such as ^67^Cu.

**Supplementary Information:**

The online version contains supplementary material available at 10.1186/s41181-024-00314-7.

## Introduction

Targeted radiopharmaceuticals for cancer imaging and radiotherapy generally possess only one single targeting vector that binds with high affinity and selectivity to the target, serving as a vehicle for the specific transport of the activity to the site of interest such as the primary tumor and metastases. A commonly used approach in the development of targeted radiopharmaceuticals to improve their pharmacokinetic profile is called multimerization; that is the introduction of multiple biomolecules as targeting vectors into the corresponding bioconjugate. If the distance between the targeting vectors is long enough to allow simultaneous binding of the homomultimeric radiotracer to two or more binding sites e.g., receptors, such compounds profit from the so-called multivalency effect that generally leads to an increased tumor uptake and prolonged retention of the corresponding radiopharmaceutical compared to their monovalent counterparts (Böhmer et al. 2021). Even if multivalency is not given, homomultimeric radiotracers may still exhibit superior properties compared to monomeric compounds, which can be explained by a higher “local concentration” of the biomolecule in close proximity of the respective target/receptor favoring rebinding, ultimately leading to a reduced dissociation rate from the target. Apart from factors such as target affinity, uptake, and retention, multimeric radiotracers often show different biodistribution profiles due to their higher molecular weights and differences in polarity compared to their monomeric congeners, offering the possibility of pharmacokinetic optimization.

One of the first targets for which multimeric radiotracers have been developed was the α_v_ß_3_ integrin receptor, which is upregulated in activated endothelial cells of tumors undergoing angiogenesis but is not expressed in normal cells and quiescent vessel cells making it a key target for the diagnosis of malignant tumors and metastases (Hood and Cheresh 2002; Sheldrake and Patterson 2009). Due to the low expression profile of the α_v_ß_3_ integrin receptor, multiple multimeric/-valent radiotracers were developed in order to increase the tumor uptake by making use of the multivalency effect (Carlucci et al. 2012; Liu 2009, 2006; Dijkgraaf et al. 2007; Garanger et al. 2006; Kok et al. 2002).

Another molecular target that is currently subject of extensive research in radiopharmaceutical development focusing on multimeric compounds is the fibroblast activation protein (FAP). FAP is overexpressed in cancer-associated fibroblasts (CAFs) of several tumor entities, such as breast, colon, and pancreatic carcinomas, making it a key target for imaging and, potentially, radiotherapy. In this respect, monomeric quinoline-based tracers (Jansen et al. 2013) that act as FAP inhibitors (FAPIs) demonstrated promising results in preclinical studies but also in clinical PET imaging in 15 different tumor entities (Kratochwil et al. 2019; Lindner et al. 2018). However, the first generation of FAPI radiotracers suffered from rapid washout from the CAFs preventing their use for targeted radiotherapy (Giesel et al. 2019; Loktev et al. 2018). Thus, efforts are now undertaken to improve the pharmacokinetics, in particular the residence time in the tumors, by designing multimeric FAPI radiotracers that comprise two or more quinoline targeting moieties (Galbiati et al. 2022; Zhao et al. 2022; Pang et al. 2023; Ballal et al. 2021; Martin et al. 2023).

Multimeric compounds have also been developed that target the prostate specific membrane antigen (PSMA), which is overexpressed on prostate carcinoma cells. Based on the metal chelator HBED-CC, the ^68^Ga-labeled homodimer PSMA-10, has been developed that showed an improved PSMA affinity, higher cell uptake, and prolonged cell surface retention compared to the monomeric PSMA-11 (Glu-CO-Lys-Ahx-HBED-CC) (Schäfer et al. 2012). However, these properties did not result in significant differences in terms of tumor and physiological uptake and clearance compared to the monomer PSMA-11 in a small-animal model (Schäfer et al. 2012). Notni and co-workers reported a dendritic molecule employing four TRAP (1,1,4,7-triazacyclononane-1,4,7-tris[methylene(2-carboxyethyl)-phosphinic acid]) chelating moieties resulting in a hexameric PSMA inhibitor (Reich et al. 2017). In competitive displacement assays with LNCaP cells, this PSMA conjugate showed excellent PSMA binding affinity accentuating the multimerization effect. In a more recent study by Zhang et al*.*, the tetrameric PSMA radiotracer DOTA-(2P-PEG_4_)_2_ showed higher cellular affinity and uptake rate than its corresponding dimeric congener translating into high and persistent tumor uptake in small-animal experiments (Zhang et al. 2023). The most advanced dimeric compound is SAR-bisPSMA comprising two Glu-CO-Lys PSMA binding motifs linked through a macrobicyclic hexamine cage sarcophagine (SAR) ligand developed by *Donnelly* and co-workers (Zia et al. 2019). In a preclinical study, the ^64^Cu-labeled SAR-bisPSMA exhibited high tumor uptake, low background, and prolonged tumor retention, even at 24 h post injection, making this bivalent agent a promising diagnostic tracer for prostate cancer (Zia et al. 2019). The ^64^Cu- and ^67^Cu-labeled SAR-bisPSMA for diagnostic PET imaging and radioligand therapy, respectively, have been and are currently part of several clinical trials (NCT04868604, NCT06056830, NCT05249127, NCT04839367).

We recently developed a chelating platform based on the macrocycle tacn (1,4,7-triazacyclononane) containing up to three additional five-membered azaheterocyclic arms (Gotzmann et al. 2016). These chelators exhibit excellent complexation properties for Cu^2+^ cations (Gotzmann et al. 2016; Guillou et al. 2019; Läppchen et al. 2018) and the imidazole-type ligands can also be used in combination with the positron-emitter ^68^Ga and the Gamma- and Auger-emitter ^111^In (Schmidtke et al. 2017; Weinmann et al. 2018). Additionally, we showed that aliphatic substituents at the non-coordinating NH of the imidazole arms of the NOTI (1,4,7-triazacyclonoane-1,4,7-tri-methylimidazole) chelator do not impact the metal binding properties allowing the straightforward introduction of additional chemical entities for bioconjugation by simple nucleophilic substitution reactions (Gotzmann et al. 2016). This allows the design of trifunctionalized chelating moieties that can serve as central structural entities for the development of multivalent/-meric radiotracers. In this regard, we designed the chelating building block NOTI-TVA (TVA = trivaleric acid) that possesses three additional carboxylic acid functionalities at the imidazole residues for conjugation of up to three targeting vectors by peptide bond formation (Martin et al. 2021). In a proof-of-concept study, we showed for a ^64^Cu-labeled homotrimeric probe targeting the α_v_ß_3_ integrin receptor that aliphatic modifications at the non-coordinating NH groups of the imidazole residues are well-tolerated with no measurable impact on the radiolabeling properties/complex stability of the NOTI-TVA scaffold (Martin et al. 2021). In line with reports on other multimeric RGD-based radiotracers, the ^64^Cu-labeled homotrimeric RGD conjugate displayed a higher binding affinity and cellular internalization than its monomeric counterpart in the α_v_ß_3_-positive U-87MG cell line (Martin et al. 2021). In ex vivo biodistribution and PET imaging studies, this ^64^Cu-labeled trimer displayed superiority over the monomer with a ~ 2.5-fold higher tumor accumulation for up to 24 h post administration, confirming the applicability of the NOTI-TVA scaffold for the design of ^64^Cu-labeled trimeric radiotracers.

In the present work, we sought to further explore the potential of the NOTI-TVA building block for the development of homotrimeric radioconjugates. In this respect, we designed a series of homotrimeric PSMA conjugates and evaluated their properties in terms of target affinity, specific cell uptake, metabolic stability and capability of delineating PSMA-positive tumors by PET imaging in LNCaP xenograft bearing mice. Additionally, the best candidate of the ^68^Ga-labeled series was used in a first clinical PET/CT imaging study.

## Results

### ***Conjugate syntheses, ***^***nat***^***Ga complexes, and radiolabeling with ***^***68***^***Ga***

**Scheme 1:**
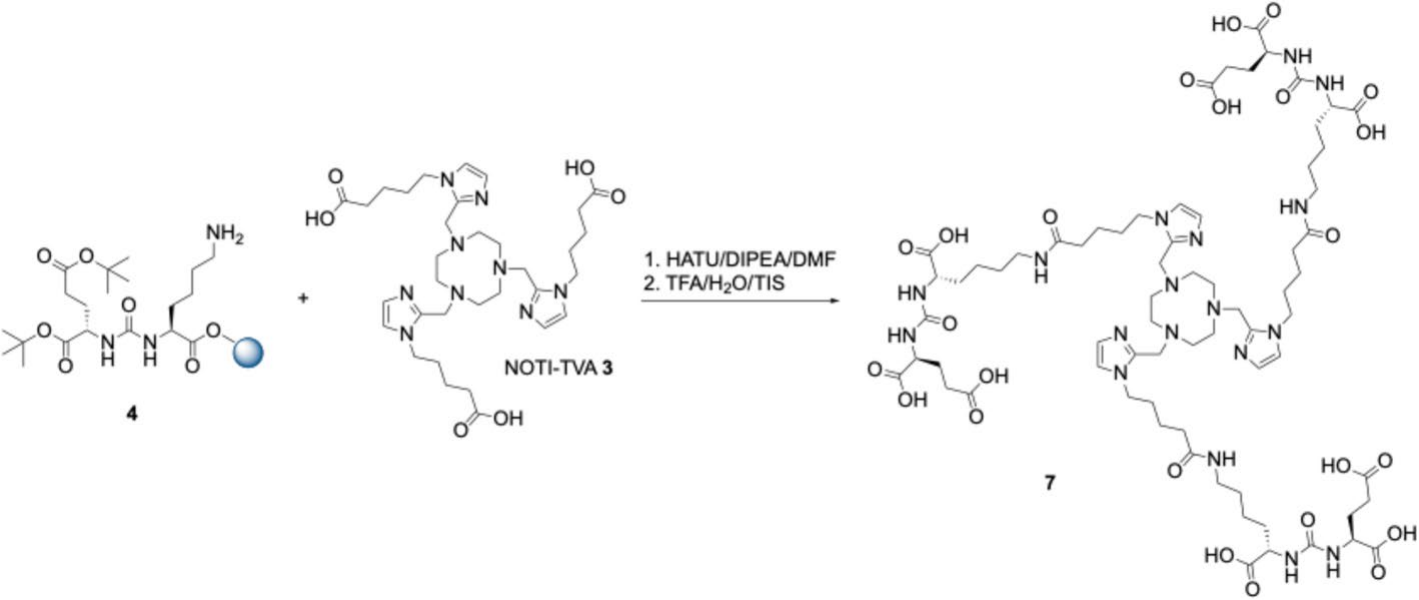
Synthesis of the homotrimeric PSMA conjugate **7** with no spacer between the Glu-CO-Lys binding motif **4** and the NOTI-TVA chelating scaffold **3**

Altogether, three different conjugates comprising different spacer entities based on the chelating building block NOTI-TVA **3** were prepared. The Glu-CO-Lys binding motif **4** was prepared first on solid support according to the literature (Stemler et al. 2021). The compound **3** was synthesized as previously reported (Martin et al. 2021). For the first compound in the series, NOTI-TVA **3** was reacted directly with the resin-bound Glu-CO-Lys binding motif **4** under standard peptide coupling conditions using HATU and DIPEA (Scheme 1). After simultaneous removal of the tBu protecting groups and cleavage from the resin using a TFA/TIS/H_2_O cocktail (95:2.5:2.5, v/v/v), the final conjugate NOTI-TVA-(PSMA)_3_ **7** with no additional spacer entity was isolated in 2.5% overall yield after purification by semipreparative RP-HPLC.

**Scheme 2:**
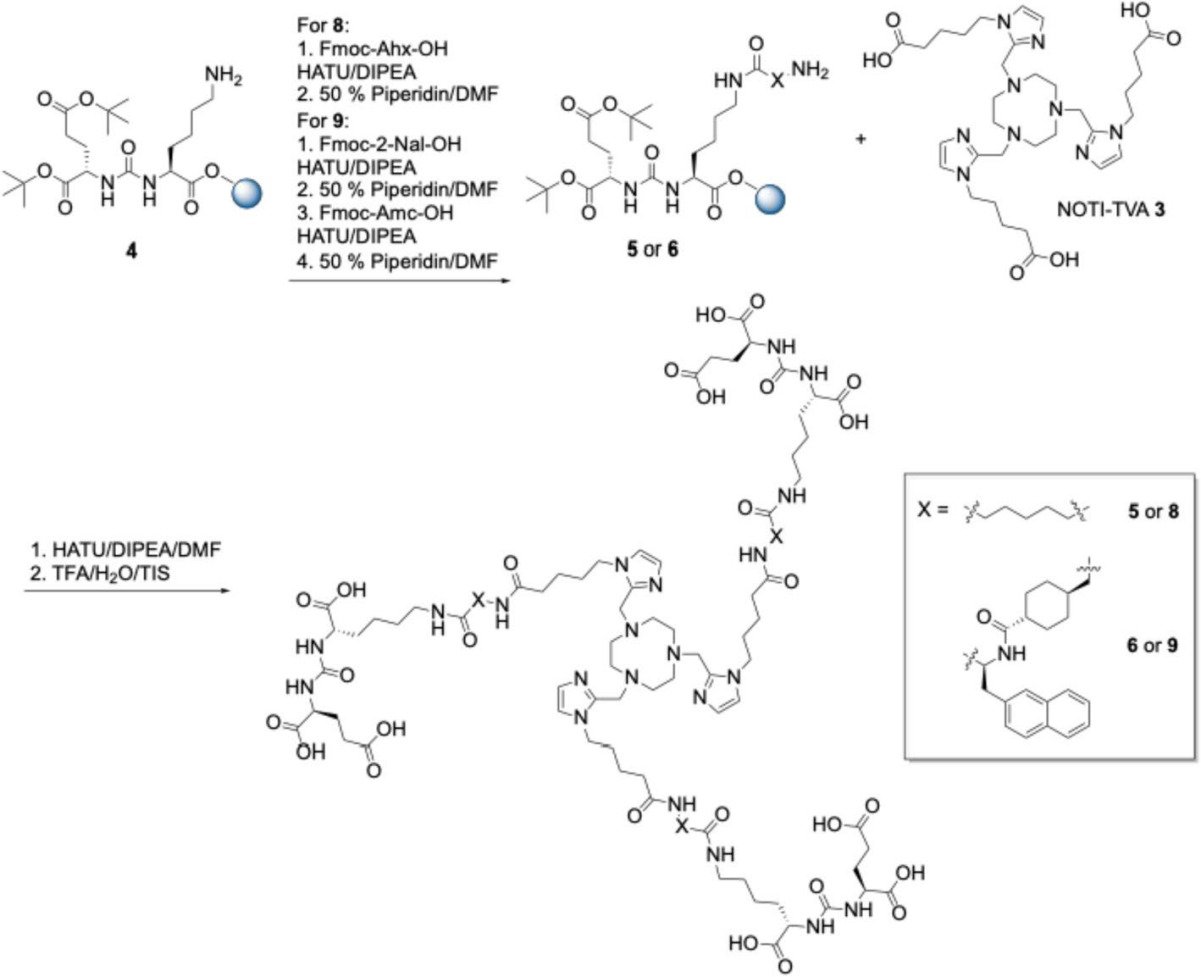
Syntheses of the homotrimeric PSMA conjugates **8** and **9** with a 6-Ahx and a Nal-Amc spacer, respectively

For the second conjugate, a 6-aminohexanoic acid spacer between the PSMA binding motif and the chelating entity was used. For this, Fmoc-6-Ahx-OH was reacted with **4** using HATU and DIPEA followed by coupling of NOTI-TVA **3** under similar conditions (Scheme 2). Subsequent cleavage from the solid support Gave the final conjugate NOTI-TVA-THA-(PSMA)_3_ **8** in 3.1% yield. The third conjugate **9** comprising the identical spacer as PSMA-617 was prepared analogously in 2.8% overall yield. All intermediates and final bioconjugates were characterized by NMR spectroscopy (where appropriate), high and low resolution electrospray mass spectrometry, and analytical HPLC. Corresponding data is provided as Supplementary Material. Of note, in the HR-MS data the *m/z* found corresponded to the Zn^2+^ adducts instead of the metal-free compounds, which can be attributed to the high affinity of the chelator to divalent zinc. This is a known phenomenon of this type of chelator and has been observed on several occasions in our laboratory. Since no Zn^2+^ adducts were found in the MS data (measured in our own laboratory) under strictly metal-free conditions, the Zn^2+^ impurities were the result of sample preparation and handling in the external MS facility underlining the high affinity of this type of ligand to Cu^2+^ and Zn^2+^ cations.

The non-radioactive ^nat^Ga complexes of **7**,** 8**, and **9** were prepared for the competitive binding experiments and for the identification of the radioactive HPLC traces of the ^68^Ga-labeled conjugates. Briefly, each conjugate was reacted with 2 equivalents of a Ga(III)(NO_3_)_3_ stock solution in sodium acetate buffer (pH 4.5) for 10 min at 95 °C. After purification using a C_18_ Sep Pak cartridge, the ^nat^Ga complexes were isolated in quantitative yields and chemical purities of > 95%.

The labeling of **7**,** 8**, and **9** with ^68^Ga was performed in a fully automated, cGMP (current good manufacturing practice) compliant process using the Eckert&Ziegler Pharmtracer module in combination with sterile single-use cassettes as previously described (Schmidtke et al. 2017). Briefly, the generator eluate (~ 400 MBq) was concentrated and purified by trapping on a cation exchange cartridge in accordance with previously reported methods (Mueller et al. 2012). Labeling of the bioconjugates was achieved in sodium acetate buffer (pH 4.5) at 95 °C for 10 min. After purification using a C_18_ Sep Pak cartridge, the products were sterile-filtered (0.22 µm) and formulated with saline. The radiochemical purities (RCPs) were > 96% and the decay corrected radiochemical yields (RCYs) were > 95%. The mean molar activities were *A*_m_ = 5.0 ± 0.2 MBq nmol^−1^ (*n* = 7) for preclinical experiments and *A*_m_ = 20.0 ± 1.3 MBq nmol^−1^ (*n* = 5) for preliminary clinical PET imaging, respectively.

### In vitro* characterization*

In order to establish a structure–activity relationship, the binding affinity of the metal-free and ^nat^Ga-complexes of the conjugates **7**,** 8**, and **9** were determined in the PSMA-positive human prostate cancer cell line LNCaP by a competitive binding assay as previously reported using [^177^Lu]Lu-PSMA-617 as the radioligand (Stemler et al. 2021). The conjugate Glu-CO-Lys-Ahx-HBED-CC (PSMA-11) was included as the control. Corresponding half maximum competitive inhibitory constants (IC_50_ values) of the metal-free conjugates **7**,** 8**, and **9** and corresponding ^nat^Ga-complexes are summarized in Table 1.

**Table 1:** Analytical and in vitro data of the investigated homotrimeric PSMA conjugates and their corresponding ^nat/68^Ga complexes

Compound	Analytical HPLC_UV/vis_/*t*_R_ min^a)^	*m/z*^b)^	RCY^c)^/%	Protein binding/%	Serum stability 1 h/% intact	Serum stability 2 h/% intact	IC_50_^d)^/nM	95% CI^e)^	Cell internalization/%
**7**	12.1	787.6 [M + 2H]^2+^	–	–	–	–	264	34.8–2004	
**8**	14.1	957.4 [M + 2H]^2+^	–	–	–	–	65	20.2–209	
**9**	23.5	861.9 [M + 3H]^3+^	–	–	–	–	5.9	0.86–40.6	
^nat/68^Ga-**7**	11.4	1639.6951 [M-2H]^+^	> 99%	63.8 ± 4.0	> 96	> 96	450	101–2010	–
^nat/68^Ga-**8**	13.4	989.9750 [M-H]^2+^	> 99%	68.2 ± 1.5	> 97	> 97	90	27.0–300	0.08 ± 0.02
^nat/68^Ga-**9**	23.1	1324.6272 [M-H]^2+^	> 96%	95.3 ± 0.3	–	–	4.8	2.4–9.8I	17.8 ± 2.5

a) Retention time, b) mass-to-charge ratio determined by LR- and HR-ESI–MS, c) radiochemical yield, d) half maximum competitive inhibitory concentration, e) CI = confidence interval

The metal-free conjugates **7**,** 8**, and **9** exhibited IC_50_ values of 264, 65, and 5.9 nM, respectively (95% confidence intervals are given in Table 1). The IC_50_ value of PSMA-11 was determined to 18.5 nM (95% confidence interval 7.5 to 45.8). The IC_50_ values of the ^nat^Ga^3+^ complexes of **7**,** 8**, and **9** were determined to 450, 90, and 4.8 nM, respectively (95% confidence intervals are given in Table 1).

**Fig. 1:**
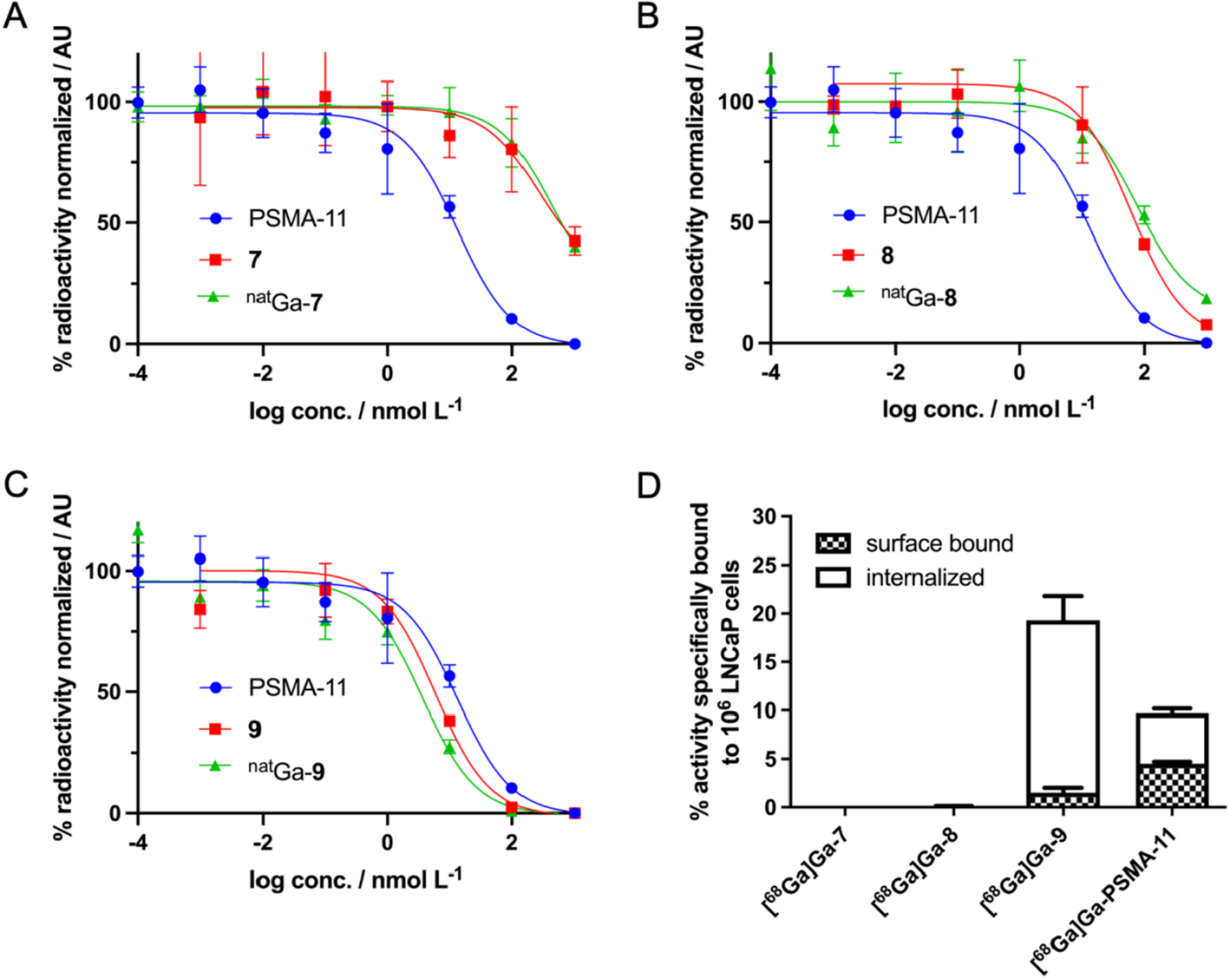
Competitive binding curves of the metal-free and ^nat^Ga complexes of **7** (**A**), **8** (**B**), and **9** (**C**) in comparison to PSMA-11. **D** Percentages of surface-bound and internalized activities of [^68^Ga]Ga-**7**, [^68^Ga]Ga-**8**, [^68^Ga]Ga-**9**, and [^68^Ga]Ga-PSMA-11 after 1 h incubation time (2.5 pmol per 10^6^ LNCaP cells)

Next, the cellular uptake of the ^68^Ga-labeled conjugates **7**,** 8**, and **9** as well as [^68^Ga]Ga-PSMA-11 was determined in PSMA-positive LNCaP cells. PSMA specificity was confirmed by blockade using the highly potent PSMA inhibitor 2-(phosphomonomethyl)pentate-1,5-dioic acid (2-PMPA) (Bařinka et al. 2012). A graphical representation of the results for 1 h incubation time is given in Fig. 1. Cell uptake of the radiolabeled low-affinity conjugates [^68^Ga]Ga-**7** and [^68^Ga]Ga-**8** was negligible, whereas the cell-surface bound and internalized fractions for [^68^Ga]Ga-PSMA-11 were 4.5 ± 0.2% and 5.2 ± 0.2%, respectively. The high PSMA affinity for **9** translated into high cell uptake of [^68^Ga]Ga-**9** with 1.5 ± 0.5% of the activity found on the cell surface and 17.8 ± 2.5% being internalized.

Incubation of the ^68^Ga-labeled conjugates **7**,** 8**, and **9** in human serum (male, AB) revealed their high metabolic stability with no significant degradation being noted for up to 2 h incubation time (Table 1). The serum protein-bound fractions of the trimeric compounds were comparatively high with about 64, 65, and 95% of ^68^Ga-labeled **7**,** 8**, and **9**.

### Small-animal PET imaging

**Fig. 2:**
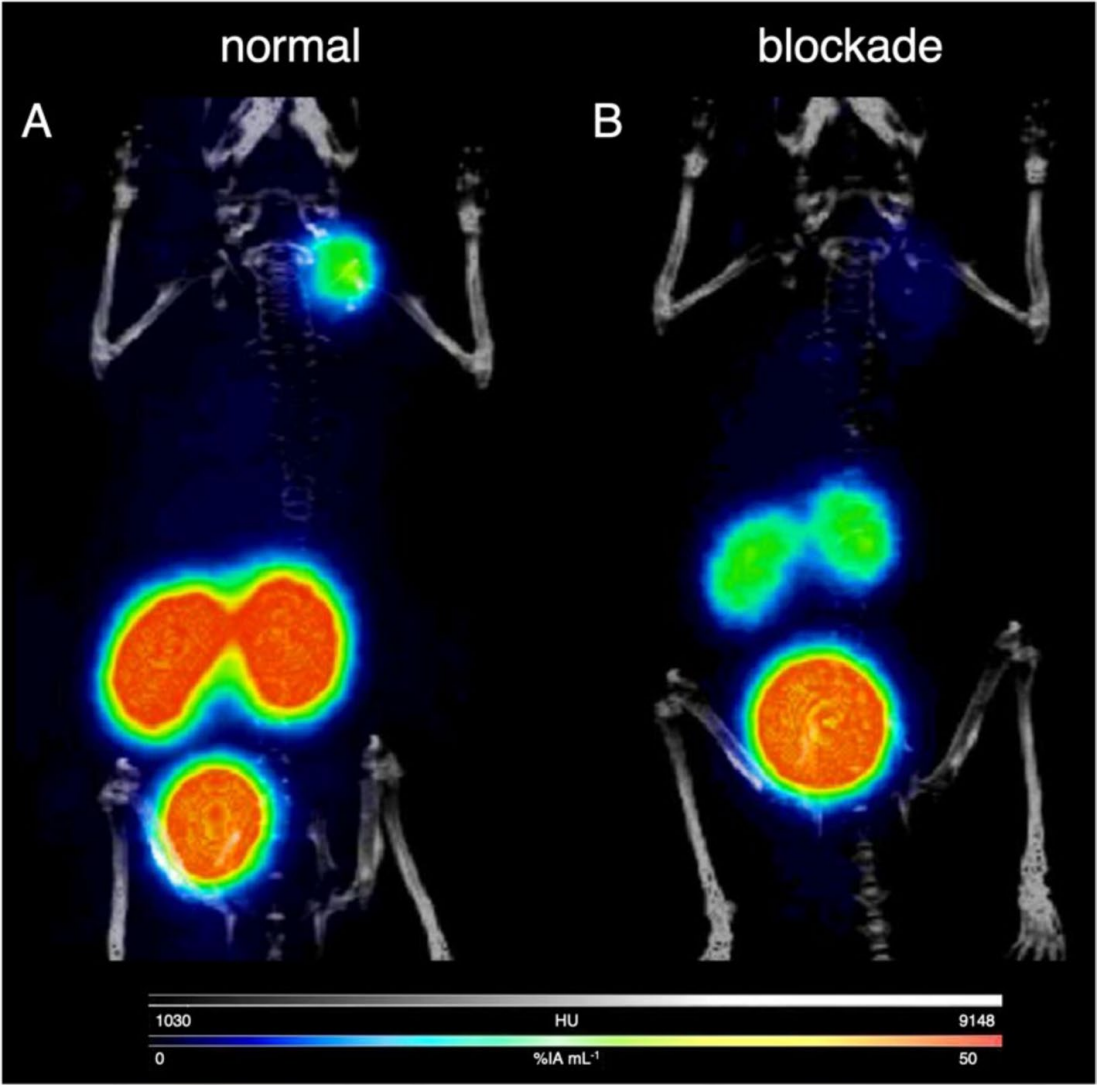
Representative coronal maximum intensity projection PET/CT images of mice bearing LNCaP xenografts injected with 1–3 MBq (250–500 pmol) of [^68^Ga]Ga-**9** at 1 h post-injection. **A** normal group; **B** blockade by co-injection of 2-PMPA (1 µmol mouse^−1^)

The capability of [^68^Ga]Ga-**9** to delineate PSMA-expressing tumors in vivo was evaluated by small-animal PET imaging at 1 and 2 h p.i. For this, mice bearing LNCaP xenografts (*n* = 3) were injected with 1–3 MBq (250–500 pmol) [^68^Ga]Ga-**9** into a tail vein and subjected to micro-PET/CT imaging at 1 h post-injection (p.i.). PSMA specificity was confirmed by co-injection of 2-PMPA (1 µmol mouse^−1^). Representative coronal maximum intensity projection (MIP) PET/CT images for 1 h p.i. are provided in Fig. 2. No significant differences were noted at 2 h p.i. LNCaP tumors were clearly visualized by [^68^Ga]Ga-**9** and only the kidneys and the bladder as the major excretory organs were visible. Image analysis revealed a tumor uptake of 16.03 ± 1.32% IA g^−1^ (% injected activity per gram), which was reduced to 3.77 ± 1.02% IA g^−1^ in the blocking group, confirming PSMA-mediated tumor accumulation. The kidney uptake was 67.86 ± 8.35% IA g^−1^ (*n* = 3) in the normal group *vs*. 19.9 ± 4.43% IA g^−1^ in the blocking group (*n* = 3). The salivary glands, which are known to express PSMA, exhibited an uptake of 2.75 ± 0.53% IA g^−1^ *vs*. 1.28 ± 0.21% IA g^−1^ in the blocking group. The blood uptake was relatively high at 1 h p.i. with 3.62 ± 0.42% IA g^−1^. Uptake in the muscles was low with 0.32 ± 0.12% IA g^−1^. The liver accumulation was 3.10 ± 0.10% IA g^−1^, indicating partial hepatobiliary excretion.

### Clinical PET imaging

**Fig. 3:**
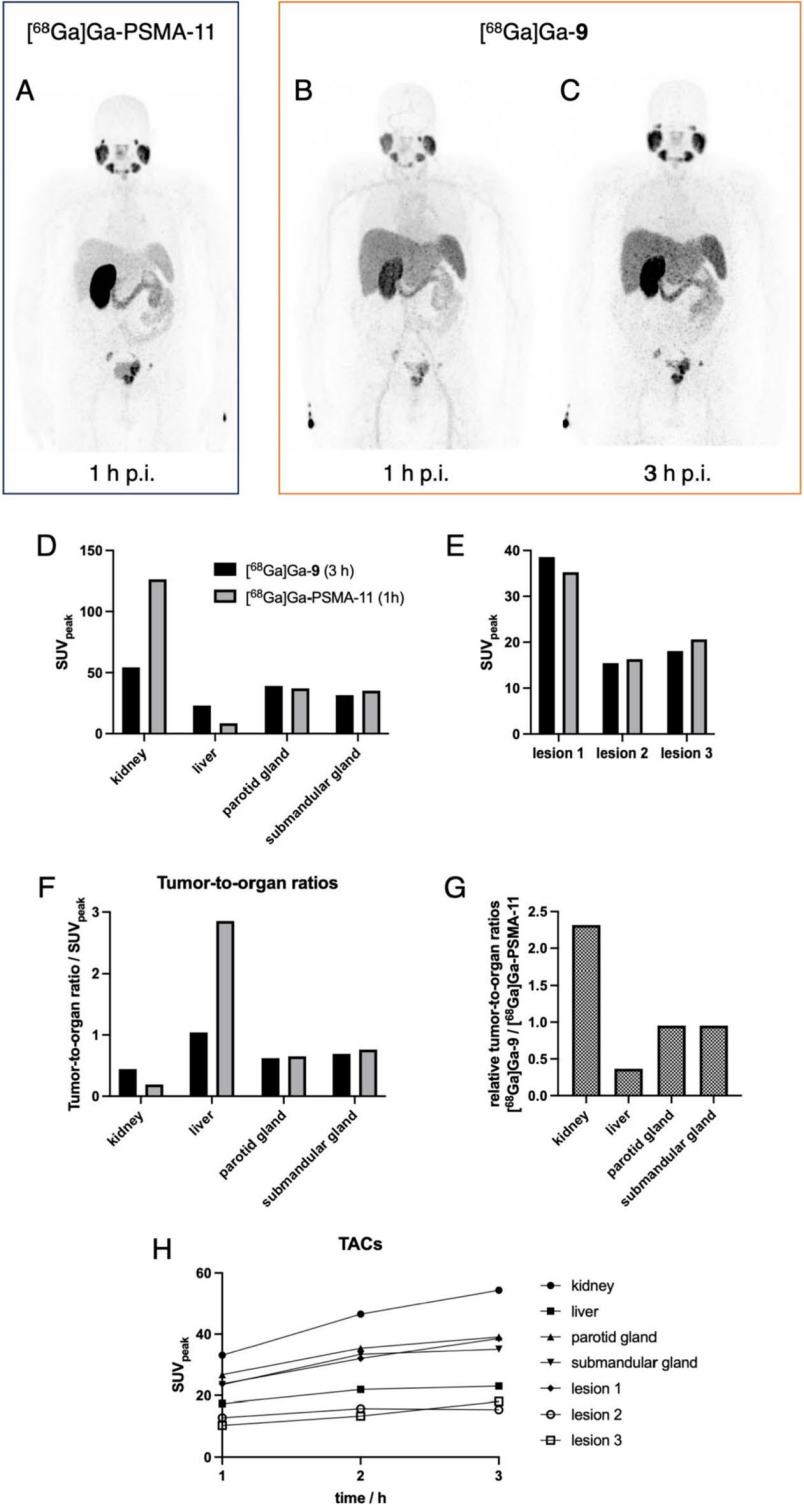
PET imaging of a 60-year old male prostate carcinoma patient with multiple lymph node metastases. **A** Coronal MIP PET image at 1 h p.i. with 113 MBq [^68^Ga]Ga-PSMA-11. **B** + **C** Coronal MIP PET images at 1 and 3 h using 107 MBq [^68^Ga]Ga-**9**, respectively. Comparison of SUV_peak_ of [^68^Ga]Ga-PSMA-11 at 1 h p.i. *vs*. [^68^Ga]Ga-**9** at 3 h p.i. **D** in healthy organs and **E** in the three target lesions (1 × prostate, 2 × lymph node metastases). **F** Comparison of corresponding tumor-to-organ ratios. **G** Relative ratio of tumor-to-organ ratios between [^68^Ga]Ga-PSMA-11 and [^68^Ga]Ga-**9**. **H** Time activity curves (TACs) for [^68^Ga]Ga-**9** at 1, 2, and 3 h p.i

The encouraging results of the PET imaging study in LNCaP xenograft bearing mice prompted us to use [^68^Ga]Ga-**9** for improved clinical staging of prostate cancer by clinical PET/CT imaging. Figure 3 shows the PET images of a patient with metastatic prostate cancer imaged with [^68^Ga]Ga-**9** in comparison to the current gold standard [^68^Ga]Ga-PSMA-11. In both scans, we identified and analyzed three target lesions (1 × prostate, 2 × lymph node metastasis) with SUV_peak_ (standardized uptake value) at 1 h p.i. of 35.3, 16.3, and 20.6 for [^68^Ga]Ga-PSMA-11 and 23.7, 12.8, and 10.3 for [^68^Ga]Ga-**9**, respectively. Figure 3B shows that a substantial amount of the trimer [^68^Ga]Ga-**9** was still in circulation at 1 h p.i. The salivary glands as an organ that physiologically expresses PSMA the SUV_peak_ in the parotid gland at 1 h p.i. were 37.0 for [^68^Ga]Ga-PSMA-11 and 26.7 for [^68^Ga]Ga-**9**, respectively. The corresponding values for the submandibular gland were 31.6 *vs*. 23.5. The liver uptake of [^68^Ga]Ga-**9** at 1 h p.i. was ~ twofold higher than that of [^68^Ga]Ga-PSMA-11 with SUV_peak_ of 17.4 and 8.5, respectively. In contrast, the accumulation in the kidneys of the trimeric conjugate at 1 h p.i. was ~ fourfold lower than that of the monomeric PSMA-11 with SUV_peak_ of 33.1 *vs*. 126.3. The time-activity curves (TACs) for [^68^Ga]Ga-**9** in Fig. 3H show that the accumulation of the trimer in healthy organs and the tumor lesions was still increasing over the investigated time frame as a consequence of its prolonged biological half-life due to binding to serum proteins. Consequently, the SUV_peak_ for the liver increased from 17.4 over 21.9 to 23.0 for 1, 2, and 3 h p.i. and the SUV_peak_ for the kidney increased from 33.1 over 46.5 to 54.4. Similarly, activity accumulation in the three lesions increased gradually over time. Considering the relative increase in the tumors and healthy organs, the activity accumulation at 3 h p.i. was slowly approaching a plateau. For example, the relative increase in liver uptake was ~ 25% between 1 and 2 h p.i. and declined to ~ 5% between 2 and 3 h p.i. Accordingly, the relative kidney uptake declined from ~ 40% to ~ 17% in the same time frame. In contrast, the relative activity accumulations in the lesions Gave mixed results with a decline from ~ 35% between 1 and 2 h p.i. to ~ 20% between 2 and 3 h p.i. for the first lesion, an increase of ~ 23% to no further uptake in the second lesion, and an initial increase from ~ 29% to another ~ 36% for the third lesion.

## Discussion

The NOTI-TVA scaffold **3** provides three carboxylic acids for bioconjugation (Scheme 1). Since the distance between the PSMA-targeting Glu-CO-Lys moiety and the chemical composition of the spacer entities can impact important parameters of a multimeric radiotracer such as target affinity, cell internalization, and biodistribution, etc., we prepared a small series of compounds in order to identify a suitable candidate for clinical translation. To study this influence, altogether three conjugates with no spacer (**7**), a 6-aminohexanoic acid (**8**), and a Nal-Amc spacer (**9**) were thus prepared. A highly intriguing finding was that the direct reaction of the NOTI-TVA scaffold **3** with the corresponding resin-bound binding motifs and their spacer entities provided solely the corresponding homotrimeric conjugates. Formation of other species, such as corresponding mono- and difunctionalized NOTI-TVA **3**, was not noted. This will allow the straightforward preparation of other homotrimeric compounds with different targeting vectors by solid phase synthesis using the NOTI-TVA building block in the future.

The competitive cell binding studies revealed a strong dependency of the binding affinity of the conjugates on the length and the chemical composition of the spacer between the Glu-CO-Lys binding motif and the NOTI chelating entity. It is known that the PSMA binding cavity has a funnel-shaped entrance tunnel of approximately 20 Å in length, and an arene binding site at the external surface of the protein (Zhang et al. 2010). The spacer of **7** is obviously too short to allow optimal accommodation of the conjugate into the PSMA binding pocket. Elongation by an additional 6-aminohexanoic acid spacer as for **8** resulted in a ~ threefold increase of affinity indicating a sufficient spacer length. Compared to PSMA-11, however, the affinity was still lower by a factor of ~ 3. The PSMA binding pocket possesses an arene-binding site close to the entrance funnel of the internal PSMA cavity (Barinka et al. 2008; Kopka et al. 2017). The clinically used radiotracers PSMA-11 and PSMA-617 do possess lipophilic aromatic ring systems (PSMA-11 in the HBED-CC metal chelator; PSMA-617 in the linker region) that interact advantageously with the arene binding site (Eder et al. 2012; Benesova et al. 2016). The lack of such lipophilic aromatic residues in **8** may explain its lower IC_50_ values compared to PSMA-11. In contrast, conjugate **9** with a Nal-Amc spacer analogous to PSMA-617 exhibited a very high PSMA affinity, underlining that not only the length but also the chemical composition of the spacer plays a crucial role for PSMA affinity. Moreover, the IC_50_ value for **9** with 5.9 nM is about threefold lower than that of PSMA-11 with 18.5 nM being indicative for a rebinding effect caused by multimerization.

While metal complexation had no significant impact on the binding affinity for ^nat^Ga-**9**, the affinity decreased significantly for ^nat^Ga-**7** and ^nat^Ga-**8** in comparison to the metal-free conjugates. This decrease for ^nat^Ga-**7** and ^nat^Ga-**8** may be explained by the steric strain that is induced upon metal binding resulting in less flexibility of the conjugates to arrange for optimal accommodation in the PSMA pocket in combination with the insufficient length and composition of the linker moieties. Contrarily, metal complexation did not significantly impact the affinity of **9** suggesting that the spacer is of sufficient length to minimize the influence of metal complexation with regards to target binding.

The in vitro experiments in human serum showed excellent metabolic stability of the ^68^Ga-labeled conjugates **7**, **8**, and **9**. But more interestingly, high binding to serum proteins was noted for all conjugates. This is an intriguing finding, in particular for [^68^Ga]Ga-**9** being almost quantitatively bound to serum proteins, in light of recent efforts to incorporate additional albumin-binding moieties into PSMA radiopharmaceuticals in order to optimize their pharmacokinetics and to achieve increased doses delivered to the tumors during radioligand therapy (Kelly et al. 2019; Iikuni et al. 2022; Tschan et al. 2022; Reissig et al. 2022; Boinapally et al. 2023). During PSMA targeted radioligand therapy using [^177^Lu]Lu-PSMA-617 more than 30% of patients may not respond to therapy, and the relapse rate remains high, which is partly attributed to poor pharmacokinetics and particularly due to insufficient dose delivery to the tumor (Kratochwil et al. 2016). For example, 50% of injected activity of [^177^Lu]Lu-PSMA-617 is excreted within 4 h of administration and by 12 h, nearly 70% of the activity is usually excreted (Kurth et al. 2018). Binding to albumin leads to a prolonged circulation of the radiotracer in the blood pool and, thus, to an increased area under the curve and, additionally, to increased tumor-to-kidney ratios in radiotherapeutic applications. Even though conjugate **9** was evaluated as diagnostic agent in this work, its intrinsic capability to bind to serum proteins makes it a promising candidate for future applications in combination with therapeutic radionuclides such as ^67^Cu.

Altogether, the in vitro evaluations allowed the identification of the homotrimeric PSMA conjugate **9** with high target affinity, cell internalization, and metabolic stability, which was a suitable candidate for further evaluations in vivo in tumor xenograft bearing mice. Small-animal PET/CT imaging using [^68^Ga]Ga-**9** in mice bearing PSMA-positive LNCaP tumor xenografts revealed high and specific tumor uptake being in line with the results of the cell experiments. The relatively high molecular weight (~ 2.6 kDa) of [^68^Ga]Ga-**9** combined with its intrinsic high serum protein binding resulted in an altered biodistribution profile compared to monomeric PSMA radiotracers. Despite experimental differences, the uptake values for [^68^Ga]Ga-**9** may carefully be compared to the biodistribution data reported for other PSMA radiotracers (Table 2) (Benešová et al. 2015; Eder et al. 2012). The tumor uptake of the homotrimeric compound [^68^Ga]Ga-**9** was ~ 1.5–twofold higher compared to [^68^Ga]Ga-PSMA-617, [^68^Ga]Ga-PSMA-11, and [^177^Lu]Lu-PSMA-617 at 1 h p.i. Significant differences between [^68^Ga]Ga-**9** and the clinically established PSMA radiotracers were also observed in the excretory organs. For example, the liver uptake of [^68^Ga]Ga-**9** was significantly higher, whereas the kidney uptake was significantly lower than those values reported for [^68^Ga]Ga-PSMA-617, [^68^Ga]Ga-PSMA-11, and [^177^Lu]Lu-PSMA-617, which can be attributed to the molecular weight of **9** and its high serum protein binding (Benešová et al. 2015; Eder et al. 2012). The differences in tumor uptake and in the excretory organs compared to the small molecule inhibitors such as PSMA-11 and PSMA-617 also influenced the tumor-to-organ ratios (Table 2). For example, the tumor-to-kidney ratio for [^68^Ga]Ga-**9** is 3–fourfold higher than that of the monomeric small molecule radiotracers, which makes the homotrimeric conjugate **9** a promising candidate for future radiotherapeutic applications.

**Table 2:** Comparison of organ uptake values and tumor-to-organ ratios of [^68^Ga]Ga-**9** compared to well-established PSMA radiotracers in LNCaP xenograft bearing mice. Data given as % injected activity per gram (% IA g^−1^) at 1 h p.i

Organ	[^68^Ga]Ga-PSMA-617^a)^	[^177^Lu]Lu-PSMA-617^a)^	[^68^Ga]Ga-PSMA-11^a)^	[^68^Ga]Ga-PSMA-11^b)^	[^68^Ga]Ga-**9**
Tumor	8.47 ± 4.09	11.20 ± 4.17	10.58 ± 4.5	7.70 ± 1.45	16.03 ± 1.32
Liver	1.17 ± 0.10	0.22 ± 0.08	–	0.87 ± 0.05	3.10 ± 0.10
Kidney	113.3 ± 24.4	137.2 ± 77.8	187.4 ± 25.3	139.4 ± 21.4	67.86 ± 8.35
Tumor-to-liver ratio	7.24	50.91	–	8.85	5.17
Tumor-to-kidney ratio	0.07	0.08	0.06	0.06	0.24

a) Taken from ref. Benešová et al. (2015), b) taken from ref. Eder et al. (2012)

Clinical PET/CT imaging using [^68^Ga]Ga-**9** confirmed its applicability for diagnostic imaging of prostate cancer. All lesions detected by the current gold standard [^68^Ga]Ga-PSMA-11 were also identified by [^68^Ga]Ga-**9** (Fig. 3). However, corresponding SUV_peak_ at 1 h p.i. were lower than those of [^68^Ga]Ga-PSMA-11 because a substantial amount of [^68^Ga]Ga-**9** was still in circulation at this time point as can be seen in Fig. 3B, which can be attributed to its high serum protein binding resulting in a prolonged retention in the blood pool. Corresponding time-activity curves (TACs) for [^68^Ga]Ga-**9** in Fig. 3H show that the accumulation of the trimer in healthy organs and the tumor lesions was still increasing over the investigated time frame of 3 h. A similar finding was observed for the salivary glands as an organ that physiologically expresses PSMA. The pronounced differences in activity accumulation in the excretory organs observed in the animal study were also reflected in clinical PET imaging (Fig. 3G). Already at 1 h p.i., [^68^Ga]Ga-**9** exhibited a ~ twofold higher liver uptake and ~ fourfold lower kidney accumulation compared to [^68^Ga]Ga-PSMA-11. From clinical PET/CT imaging study, it can also be concluded that the ideal time point for diagnostic imaging using [^68^Ga]Ga-**9** is 3 h p.i. (Fig. 3C).

For a direct comparison with the current gold standard, the uptake values for [^68^Ga]Ga-**9** at 3 h p.i. may carefully be compared with those of [^68^Ga]Ga-PSMA-11 at 1 h p.i., which is the optimal time point for diagnostic PET imaging using this low molecular weight compound (Fendler et al. 2023). As can be seen in Fig. 3D + E, the SUV_peak_ of both radiotracers for the tumor lesions and salivary glands, as organs that physiologically express PSMA, were comparable, while significant differences were noted for the excretory organs. Of note, the higher activity accumulation in PSMA-positive cells/tumors observed in vitro and in vivo in corresponding animal studies was not observed in this preliminary clinical imaging study similar to findings of other multimeric PSMA-targeting radiotracers (Schäfer et al. 2012). This may be attributed to the prolonged blood circulation and, thus, still ongoing accumulation in PSMA-expressing organs and tumors. Evaluation in larger patient cohorts, also in combination with longer lived radionuclides, in the future are necessary to gain further insights. The pronounced differences in biodistribution between [^68^Ga]Ga-PSMA-11 and [^68^Ga]Ga-**9** are also reflected in the corresponding tumor-to-organ ratios (Fig. 3F) and the relative tumor-to-organ ratios (Fig. 3G). Summarizing, the higher molecular weight and its high serum protein binding of the trimeric compound translated into distinct differences in activity accumulation for [^68^Ga]Ga-**9**.

## Conclusions

In the present work, we identified a homotrimeric PSMA conjugate based on the NOTI chelating platform with high PSMA affinity and high PSMA-mediated uptake into LNCaP cells. Due to its relatively high molecular weight compared to the current clinical gold-standard monomeric PSMA-11 and its intrinsically high binding to serum proteins, this radioconjugate exhibited extended circulation in the blood pool and a different biodistribution profile with lower kidney and higher liver uptake than PSMA-11 in animal studies as well as in preliminary PET/CT imaging in a prostate carcinoma patient. To elucidate its biodistribution at later time points, further studies in combination with longer-lived radionuclides, such as ^64^Cu, are warranted. Finally, its prolonged biological half-life and its improved tumor-to-kidney ratio make this homotrimeric conjugate a potential candidate for future radiotherapeutic applications in combination with therapeutic radionuclides such as ^67^Cu.

## Material and methods

### Chemicals

Chemicals and solvents of analytical grade were purchased from Sigma Aldrich, Merck, Iris Biotech, DEUTERO, Carl Roth, Honeywell, and TCI and used as received. The 2-CTC resin was obtained from Carbolution (St. Ingbert, Germany). [^68^Ga]GaCl_3_ was obtained by eluting a GalliaPharm generator (Eckert&Ziegler AG, Germany). NMR spectra were recorded on a Bruker *Avance II WB* (^1^H 400 MHz, ^13^C 101 MHz), a Bruker *Avance III HD* (^1^H 300 MHz, ^13^C 75 MHz,) or a Bruker *DPX* (^1^H 200 MHz, ^13^C 50 MHz) at 298 K. NMR solvents were d_6_-DMSO and d_4_-MeOD. Spectra were calibrated on solvent signals (7.26 ppm for CDCl_3_, 3.33 ppm for d_4_-MeOD) (Gottlieb et al. 1997). Chemical shifts are given in parts per million (ppm) and are reported relative to trimethylsilane (TMS). Coupling constants are reported in hertz (Hz). The multiplicity of the NMR signals is described as follows: s = singlet, d = duplet, t = triplet, q = quartet, m = multiplet. Low resolution electrospray ionisation mass spectrometry (( +)LR-ESI–MS) was performed on an Advion expressionCMS mass spectrometer (Ithaca, NY, USA). High resolution mass spectrometry (( +)-HR-ESI–MS) was performed on a Thermo Scientific Exactive mass spectrometer (Waltham, MA, USA). Samples were lyophilized using a Christ Alpha 1–2 LD plus lyophilizer (Osterode am Harz, Germany). All instruments measuring radioactivity were calibrated and maintained in accordance with previously reported routine quality-control procedures (Zanzonico 2009). Radioactivity was measured using an ISOMED 2010 activimeter (Nuklear-Medizintechnik, Dresden, Germany). For accurate quantification of radioactivity, experimental samples were counted for 1 min on a calibrated Perkin Elmer (Waltham, MA, USA) 2480 Automatic WizardGamma Counter by using a dynamic energy window of 400–600 keV forGallium-68 (511 keV emission). Statistical analyses (Student’s t-test, confidence interval 95%) were performed using Graphpad Prism Version 7.0.

### Product purification

#### Reversed-phase semi-preparative HPLC

Semi-preparative RP-HPLC was performed on a Knauer Smartline 1000 HPLC system in combination with a Macherey Nagel VP 250/21 Nucleosil 120–5 C_18_ column at a detection wave length of 220 nm and a flow rate of 12 mL min^−1^. The solvent system was A = H_2_O (0.1% TFA) and B = acetonitrile (0.1% TFA). Gradient 1: 0–40 min 5% to 60% B, 40–45 min 80% B, 45–48 min 80% B, 48–50 min 5% B. Gradient 2: 0–1 min 5% B, 1–3 min 12% B, 3–30 min 30% B, 30–32 min 5% B. Gradient 3: 0–1 min 5% B, 1–3 min 40% B, 3–30 min 50% B, 30–32 min 5% B.

#### Normal-phase flash chromatography

NP flash chromatography was carried out on a Biotage Isolera Prime system (Uppsala, Sweden) using a silica gel column (SNAP KP-Sil 50 g). The solvent system was A = n-hexane and B = ethyl acetate. The flow rate was 50 mL min^−1^ and the detection wave length was adjusted to 280 nm. Gradient: 3 cartridge volumes (CVs) 0% B, 3 CVs from 0 to 100% B, 4 CVs 100% B.

#### Reversed-phase flash chromatography

For RP flash chromatography, a SNAP Ultra C_18_ 30 g cartridge was used with a flow rate of 25 mL min^−1^. The solvent system was A = H_2_O (0.1% TFA) and B = acetonitrile (0.1% TFA). Gradient: 2 CVs 0% B, 6 CVs from 0 to 100% B, 2 CVs 100% B.

### Analytical HPLC

Analytical HPLC measurements were conducted on a 1260 Infinity HPLC system (Agilent Technologies, USA) (UV detection at 280 nm) and a Raytest Ramona radiation (detection window 100–900 keV) detector (Raytest GmbH, Straubenhardt, Germany) in series at a flow rate of 1 mL min^−1^. The solvent system was A = H_2_O (0.1% TFA) and B = acetonitrile (0.1% TFA). Gradient 4: Phenomenex RP 12 column (Phenomenex Jupiter 4 μm Proteo 90 Å LC 250 × 4.6 mm) 0–1 min 5% B, 1–25 min 50% B, 25–27 min 95% B, 27–29 min 95% B, 29–32 min 5% B, 32–35 min 5% B.

### Bioconjugate syntheses

#### Methyl 5-(2 formyl-1H-imidazol-1-yl)pentanoate (1)

Imidazole-2-carboxyaldehyde (3.0 g, 3.31 mmol), potassium carbonate (8,62 g, 6.26 mmol), and methyl-2-bromovalerate (5.34 mL, ρ = 1, 363 g mL^−1^, 3.76 mmol) were added to 30 mL acetonitrile. The reaction mixture was heated at 45 °C for 24 h. The mixture was filtered and the brown filtrate was concentrated by rotary evaporation to a total volume of ~ 5 mL. The product was purified after NP flash chromatography. Fractions containing the product were combined and the solvents removed by rotary evaporation. Yield: 4.03 g (19.1 mmol, 61.2%). ^1^H NMR (CDCl_3_): δ 9.76 (s, 1H), 7.24 (d, *J* = 1.88 Hz, 1H), 7.14 (d, *J* = 1.9 Hz,1 H), 4.36 (t, *J* = 7.4 Hz, 2H), 3.62 (s, 3H), 2.31 (t, *J* = 7.0 Hz, 2H), 1.78 (m, 2H), 1.61 (m, 2H). HR-ESI( +)-MS: *m/z* calc. for C_10_H_15_N_2_O_3_, 211.1083 [M + H]^+^, found: 211.1075. Analytical HPLC (gradient 4): *t*_R_ = 9.2 min, purity > 99%.

#### Trimethyl-5,5’,5″-(((1,4,7-triazonane-1,4,7-triyl)-tris(methylene))-tris(1H-imidazole-2,1-diyl))-tripentanoate (2)

The macrocycle 1,4,7-triazacyclononane (0.20 g, 1.55 mmol) and compound **1** (1.71 g, 7.74 mmol) were dissolved in 10 mL THF and heated at 70 °C for 24 h. During the reaction, the solution adopted a yellowish color. After cooling to r.t., sodium triacetoxyborohydride (2.0 g, 9.43 mmol) was added stepwise. After 5 h, 50 mL MeOH were added and the solvents removed under reduced pressure by rotary evaporation. The resulting yellow oil was dissolved in 10 mL of a H_2_O/ACN (2:1, v/v) and the pH adjusted to pH 2–3 by addition of trifluoroacetic acid (TFA). The crude product was purified by RP flash chromatography to give compound **2** as a yellowish colored oil. Yield: 1.044 g (1.47 mmol, 94.6%). ^1^H NMR (MeOD): δ 7.61 (d, *J* = 1.44 Hz, 3H), 7.54 (d, *J* = 1.44 Hz, 3H), 4.37 (s, 6H), 4.18 (t, *J* = 5.86 Hz, 6H), 3.65 (s, 9H), 3.13 (s, 12H), 2.40 (t, *J* = 5.74 Hz, 6H), 1.82 (m, 6H), 1.61 (m, 6H). HR-ESI( +)-MS: *m/z* calc. for C_36_H_58_N_9_O_6_, 712.4510 [M + H]^+^, found: 712.4506. Analytical HPLC (gradient 4): *t*_R_ = 16.2 min, purity > 99%.

#### 5’,5″-(((1,4,7-triazonane-1,4,7-triyl)-tris-(methylene))-tris-(1H-imidazole-2,1-diyl))-tripentanoic acid (3)

Compound **2** (100 mg, 0.14 mmol) was dissolved in 5 mL H_2_O/TFA (1:1, v/v) and heated at 95 °C for 24 h. The solvents were removed by rotary evaporation and the product finally dried in vacuum to obtain compound **3**. Yield: 93.6 mg (0.14 mmol, > 99%). ^1^H NMR (d_4_-MeOD): δ 7.61 (d, *J* = 1.68 Hz, 3H), 7.54 (d, *J* = 1.72 Hz, 3H), 4.42 (s, 6H), 4.23 (t, *J* = 7.3, 6H), 3.15 (s, 12H), 2.40 (t, *J* = 7.06, 6H), 1.90 (m, 6H), 1.64 (m, 6H). HR-ESI( +)-MS: *m/z* calc. for C_33_H_52_N_9_O_6_ [M + H]^+^, 670.4041, found: 670.4042. Analytical HPLC (gradient 4): *t*_R_ = 10.5 min, purity > 99%.

#### Solid phase synthesis of Glu-CO-Lys PSMA binding motif (4)

The PSMA binding motif was prepared according to the literature (Eder et al. 2012). Briefly, Fmoc-Lys-(Alloc)-OH was immobilized on 2-chlorotritylresin. The Fmoc group of the immobilized Fmoc-Lys(Alloc)-OH (350 mg, 0.1 mmol) was further removed by a mixture of piperidine/N,N-dimethylformamide (DMF) (1:4, v/v). The amino acid H-Glu-(OtBu)-OtBu HCl (874 mg, 3 mmol) was reacted with bistrichloromethyl carbonate (296 mg, 1 mmol) at 0 °C. The resin bound lysine was added to the isocyanate reagent and stirred for 16 h at ambient temperature. Finally, the alloc protection-group of the lysine was removed by the catalyst tetrakis(triphenylphosphine) palladium(0) to obtain the resin-bound PSMA binding motif **4**. To confirm completion of the reaction, an aliquot of resin-bound PSMA binding motif **4** was reacted for 90 min at r.t. with a cleavage cocktail of TFA/H_2_O/TIS (95:2.5:2.5, v/v/v). After precipitation in 50 mL ice-cold diethylether and subsequent washing, the centrifuged pellet was analyzed without further purification. Yield of 0.2 mmol resin: 14.18 mg (0.044 mmol, 22.2%). ^1^H NMR (D_2_O): δ 4.20 (m, 2H), 2.99 (t, 2H), 2.49 (t, 2H), 2.15 (m, 1H), 1.96 (m, 1H), 1.86 (m, 1H), 1.72 (m, 1H), 1.68 (m, 2H), 1.45 (m, 2H). LR-ESI( +)-MS: *m/z* calc. for C_12_H_22_N_3_O_7_ [M + H]^+^ 320.1, found: 320.1 (100%, z = 1); Analytical HPLC (gradient 4): *t*_R_ = 4.2 min, purity > 98%.

#### Introduction of Ahx linker to the resin-bound PSMA binding motif 4 (5)

Compound **4** (0.3 mmol, 360 mg) was first agitated in 5 mL DMF for 30 min. Meanwhile, Fmoc-6-Ahx-OH (455.34 mg, 1.2 mmol), DIPEA (420 μL, 2.4 mmol) and HATU (410 mg, 1.08 mmol) were mixed in 2 mL DMF and then added to the resin. After 90 min under rotation, the resin was washed with DMF (6 × 5 mL) followed by the removal of the Fmoc protecting group using 3 × 5 mL of piperidine/N,N-dimethylformamide (DMF) (1:4, v/v) for 5, 10, and 30 min, respectively, followed by washing the resin 6 × 5 mL DMF.

#### Introduction of Nal and Amc linkers to the resin-bound PSMA binding motif 4 (6)

Compound **6** was prepared according to the procedure described for compound **5**. Briefly, compound **4** (0.3 mmol, 360 mg) was first reacted with Fmoc-2-Nal-OH (525 mg, 1.2 mmol). Under similar conditions, trans-4-(Fmoc-aminomethyl) cyclohexane carboxylic acid (455.34 mg, 1.2 mmol) was conjugated to obtain the resin-bound **6** after removal of the Fmoc protecting group and washing steps as described for compound **5**.

#### (2S,2'S,2''S)−2,2',2''-(((((1S,1'S,1''S)-((5,5',5''-(((1,4,7-triazonane-1,4,7-triyl)tris(methylene))tris(1H-imidazole-2,1-diyl))tris(pentanoyl))tris(azanediyl))tris(1-carboxypentane-5,1-diyl))tris(azanediyl))tris(carbonyl))tris(azanediyl))triglutaric acid (7)

For the synthesis of compound **7**, the resin-bound PMSA binding motif **4** (35 mg, 0.03 mmol) was utilized, which was swelled in 10 mL DMF for 30 min. In the meantime, compound **3** (81.1 mg, 0.121 mmol) was mixed with HATU (91.2 mg, 0.24 mmol) and DIPEA (65 μL, 0.36 mmol) in 1.8 mL DMF. After addition to the resin **4**, reaction was allowed to proceed for 90 min at r.t. during that time, the pH of the reaction was kept at pH 8–10 by adding DIPEA. After completion of the reaction, the resin was filtered off and washed 6 × 5 mL of DMF, DCM and diethylether, respectively. The final product **7** was obtained by reacting the resin with a TFA/TIS/H_2_O (95/2.5/2.5, v/v/v) cocktail for 90 min followed by precipitation in ice-cold diethylether. After the centrifugation, the pellet was dissolved in water and further purified by semi-preparative RP-HPLC. The crude product was purified first using gradient 1 (*t*_R_ = 15.0 min). A second chromatographic purification was performed using gradient 2 (*t*_R_ = 17.0 min). Yield: 1.71 mg (1.09 µmol, 2.5%). HR-ESI(-)-MS: *m/z* calc. for C_69_H_103_N_18_O_24_Zn [M-5H]^3−^ 1631.6685, found: 543.8901 (100%, z = 3); *m/z* calc. for C_69_H_104_N_18_O_24_Zn [M-6H]^4−^ 1630.6607, found: 407.6659 (90.7%, z = 4). LR-ESI( +)-MS: *m/z* calc. for C_69_H_110_N_19_O_24_ [M + 2H]^2+^ 787.4, found: 787.6 (100%, z = 2); Analytical HPLC (gradient 4): *t*_R_ = 12.1 min, purity > 99%.

#### (3S,3'S,3''S,7S,7'S,7''S)−24,24',24''-(((1,4,7-triazonane-1,4,7-triyl)tris(methylene))tris(1H-imidazole-2,1-diyl))tris(5,13,20-trioxo-4,6,12,19-tetraazatetracosane-1,3,7-tricarboxylic acid) (8)

For the preparation of compound **8**, the Ahx modified resin **5** (34 mg, 0.03 mmol) was reacted with compound **3**, HATU (91.2 mg, 0.24 mmol) and DIPEA (65 μL, 0.36 mmol) in 1.8 mL DMF according to the procedure described for compound **7**. After cleavage from the resin with a TFA/TIS/H_2_O (95/2.5/2.5, v/v/v) cocktail for 90 min followed by precipitation in ice-cold diethylether, the crude product was purified by semi-preparative RP-HPLC using gradient 1 (*t*_R_ = 22 min). No differences in yield were noted between both synthetic routes. Yield: 2.61 mg (1.37 µmol, 3.1%). HR-ESI(-)-MS: *m/z* calc. for C_87_H_136_N_21_O_27_Zn [M-5H]^3−^ 1970.9205, found: 656.9741 (49.4%, z = 3); *m/z* calc. for C_87_H_135_N_21_O_27_Zn [M-6H]^4−^ 1969.9127, found: 492.4789 (91.3%, z = 4); *m/z* calc. for C_87_H_134_N_21_O_27_Zn [M-6H]^5−^ 1968.9049, found: 393.7816 m*/z* (82.7%, z = 5). LR-ESI( +)-MS: *m/z* calc. for C_87_H_142_N_21_O_27_ [M + 2H]^2+^ 957.1, found: 957.4 (100%, z = 2); Analytical HPLC (gradient 4): 14.1 min, purity > 99%.

#### (3S,3'S,10S,10'S,14S,14'S)−1,1'-((((5,5'-(((7-((1-(5-(((4-(((R)−1-(((S)−5-carboxy-5-(3-((S)−1,3-dicarboxypropyl)ureido)pentyl)amino)−3-(naphthalen-2-yl)−1-oxopropan-2-yl)carbamoyl)cyclohexyl)methyl)amino)−5-oxopentyl)−1H-imidazol-2-yl)methyl)−1,4,7-triazonane-1,4-diyl)bis(methylene))bis(1H-imidazole-2,1-diyl))bis(pentanoyl))bis(azanediyl))bis(methylene))bis(cyclohexane-4,1-diyl))bis(3-(naphthalen-2-ylmethyl)−1,4,12-trioxo-2,5,11,13-tetraazahexadecane-10,14,16-tricarboxylic acid) (9)

The resin-bound Nal-Amc-modified PSMA binding motif **6** (0.03 mmol, 44.4 mg) was swelled in 10 mL DMF for 30 min. In the meantime, compound **3** (82.2 mg, 0.122 mmol), HATU (91.2 mg, 0.24 mmol) and DIPEA (65 μL, 0.36 mmol) were mixed in 1.8 mL DMF. Further steps were carried out as described for compound **8**. The crude product was purified first using gradient 1 (*t*_R_ = 34.0 min). A second chromatographic purification was performed using gradient 3 (*t*_R_ = 15.0 min). Yield: 3.23 mg (1.25 µmol, 2.8%). HR-ESI(-)-MS: *m/z* calc. for C_132_H_175_N_24_O_30_Zn [M-5H]^3−^ 2640.2197, found: 880.4084 (20.6%, z = 3); *m/z* calc. for C_132_H_174_N_24_O_30_Zn [M-6H]^4−^ 2639.2119, found: 660.0543 (64.8%, z = 4); *m/z* calc. for C_132_H_173_N_24_O_30_Zn [M-7H]^5−^ 2638.2040, found: 528.0419 (69.2%, z = 5). LR-ESI( +)-MS: *m/z* calc. for C_132_H_183_N_24_O_30_ [M + 3H]^3+^ 861.5, found: 861.9 (100%, z = 3); Analytical HPLC (gradient 4): *t*_R_ = 23.5 min, purity > 99%.

### ***Automated***^***68***^***Ga-radiolabeling of homotrimeric conjugates 7, 8, and 9***

Radiolabelling of **7**, **8**, and **9** with [^68^Ga]GaCl_3_ was accomplished by using the Modular-Lab PharmTracer automated synthesis module (Eckert&Ziegler, Berlin, Germany) in combination with sterile single-use cassettes as previously described (Schmidtke et al. 2017). The ^68^Ge/^68^Ga generator provided an activity of ~ 400 MBq. An amount of 80 nmol of **7**,** 8**, and **9** was used per labelling. The radiochemical purities (RCPs) were > 96% and the decay corrected radiochemical yields (RCYs) were > 95% for all compounds. The mean molar activities were *A*_m_ = 5.0 ± 0.2 MBq nmol^−1^ (*n* = 7) and *A*_m_ = 20.0 ± 1.3 MBq nmol^−1^ (*n* = 5) for preclinical and clinical studies, respectively. Analytical radio-RP-HPLC (gradient 4): *t*_R_ ([^68^Ga]Ga-**7**) = 12.1 min, *t*_R_ ([^68^Ga]Ga-**8**) = 14.4 min, *t*_R_ ([^68^Ga]Ga-**9**) = 25.2 min.

### ***Preparation of non-radioactive***^***nat***^***Ga complexes of homotrimeric conjugates 7, 8, and 9***

Non-radioactive reference compounds of **7**,** 8**, and **9** were prepared by reacting 500 µg of the corresponding conjugate with 2 equivalents of Ga(III)(NO_3_)_3_ from a freshly prepared aqueous stock solution (4 µmol mL^−1^) in 500 μL sodium acetate buffer (pH 4.5). In case of compund **9**, additional 250 µL of ethanol were added to the reaction mixture to avoid precipitation of the precursor. Each mixture was vortexed and heated for 10 min at 95 °C. After cooling to r.t., the mixture was purified by passing through a C_18_ Sep Pak cartridge (preconditioned with each 10 mL of ethanol and water). The cartridge was rinsed with water (10 mL) and the labeled compounds were eluted with ethanol (1 mL). The products were freeze-dried and stock solutions of 1 nmol μL^−1^ of the ^nat^Ga-labeled conjugates were prepared by redissolving the powder in water (^nat^Ga-**7** and ^nat^Ga-**8**) or in an ethanol/water mixture (2:1, v/v) (^nat^Ga-**9**). All compounds were obtained in quantitative yields.

^**nat**^**Ga-7.** HR-ESI( +)-MS: [M-2H]^+^, *m/z* calc for C_69_H_106_N_18_O_24_Ga 1639.6884, found: 1639.6951 (z = 1); *m/z* calc. for C_69_H_107_N_18_O_24_Ga 1640.6962, found: 820.3484 (z = 2). Analytical HPLC (gradient 4): *t*_R_ = 11.4 min, purity > 95%.

^**nat**^**Ga-8.** HR-ESI( +)-MS: [M-H]^2+^, *m/z* calc. for C_87_H_140_O_27_N_21_Ga 989.9742, found: 989.9750 (z = 2); *m/z* calc. for C_87_H_141_O_27_N_21_Ga 1980.9561, found: 660.3177 (z = 3). Analytical HPLC (gradient 4): *t*_R_ = 13.4 min, purity > 95%.

^**nat**^**Ga-9.** HR-ESI( +)-MS: [M-H]^2+^, *m/z* calc. for C_132_H_179_N_24_O_30_Ga, 1324.6237, found: 1324.6272 (z = 2); *m/z* calc. for C_132_H_180_O_30_N_24_Ga 2650.2553, found: 883.4186 (z = 3). Analytical HPLC (gradient 4): *t*_R_ = 23.1 min, purity > 99%.

### Serum protein binding

To determine the protein bound fraction of each trimeric radiotracer, 100 μL of the radiolabeling solution of the corresponding ^68^Ga-labeled conjugate were added to preheated (37 °C) 1000 μL human serum (male, AB, Sigma Aldrich, USA) in triplicates. Samples were incubated for 1 h at 37 °C. After cooling to r.t., the samples were centrifuged utilizing molecular cutoff (30 kDa) centrifuge tubes at 4 G for 5 min at 4 °C. The filters were additionally rinsed with PBS buffer (2 × 100 μL). The background subtracted activity in the filters and the filtrates were determined in a Gamma counter and the percentage of protein bound fractions were calculated.

### Serum stability measurements

100 μL of the radiolabeling solution of each ^68^Ga-labeled tracer were added to preheated (37 °C) 1000 μL human serum (male, AB, Sigma Aldrich, USA) in triplicates and incubated at 37 °C. At 1 and 2 h, 100 µL aliquots were taken, mixed with 100 µL ice-cold acetonitrile, and centrifuged at 4 G for 5 min at 4 °C. The corresponding filtrates were kept on ice until HPLC analysis. The supernatants were then analyzed by radio-RP-HPLC and fractions of intact compounds were calculated from the HPLC chromatograms. (gradient 4,Gamma-ray detector: 100–900 keV, injection volume: 20 μl). *t*_R_ ([^68^Ga]Ga-**7**) = 12.1 min, *t*_R_ ([^68^Ga]Ga-**8**) = 14.4 min, *t*_R_ ([^68^Ga]Ga-**9**) = 25.2 min.

### Cell culture

In vitro experiments were conducted with PSMA-positive prostatic adenocarcinoma LNCaP cell line (ATCC CRL-1740). The cells were cultured in RPMI1640 GlutaMAX medium supplemented with 10% fetal bovine serum, 1% 10,000 U mL^−1^ penicillin and 10,000 U mL^−1^ streptomycin, 1% sodium-pyruvate 100 mM in a cell incubator at 37 °C under 5% carbondioxide atmosphere.

### Competitive binding assay

The competitive binding assay was carried out using a MultiScreen®HTS Vacuum Manifold system (Merck, Germany) with 96-well filter plates. Serial dilutions of the metal-free conjugates and corresponding ^nat^Ga complexes were prepared to obtain 7 final concentrations ranging from 0 to 1000 nM for each compound. PSMA-11 was also tested as control. Experiments were performed twice in triplicate for each compound. Freshly prepared ^177^Lu-labeled PSMA-617 was used as the radioligand. A volume of 1 μL of [^177^Lu]Lu-PSMA-617 stock solution (2.83 μM) was added into each tube of the dilution series (radioligand concentration 3.14 nM). For the experiment, 10^5^ LNCaP cells were seeded into each well. The final volume of each well was 150 μL (100 μL cells in medium + 50 μL inhibitor in PBS). The experiment was incubated for 2 h at r.t. in a plate shaker. Then, the supernatants were removed by vacuum. Wells were furthermore washed out with ice-cold PBS (3 × 200 μL, pH 7.4). The filters were punched into tubes for the subsequent quantification of the activity in a Gamma-counter. A nonlinear regression algorithm was applied by the software GraphPad Prism to determine the specific inhibitory concentrations of 50% for the tested radiotracers.

### Cell internalization

LNCaP cells were seeded (10^6^ cells well^−1^) in poly-L-lysine coated 6-well plates 24 h before the experiment. Experiments were performed twice in triplicate for each compound. The radiolabeling solutions of [^68^Ga]Ga-**7**, [^68^Ga]Ga-**8**, [^68^Ga]Ga-**9**, and [^68^Ga]Ga-PSMA-11 were first diluted with PBS (pH 7.4) to a concentration of 1 nmol L^−1^. 100 µL of this dilution were added to 3900 µL PBS (pH 7.4) to obtain final stock solutions of each radiolabeled compound (0.025 nmol mL^−1^). For blocking, a stock solution of the potent PSMA inhibitor 2-PMPA with a concentration of 25 µmol L^−1^ was prepared.

On the day of the experiment, the medium was replaced by fresh preheated medium (1.4 mL w/o blocking, 1.3 mL with blocking) and the cells incubated for 1 h at 37 °C. Next, the blocking solution was added (100 μL, 2500 pmol) to the corresponding wells. Finally, the corresponding radiolabeled conjugate (100 μL, 2.5 pmol) was added to a final assay volume of 1.5 mL well^−1^. After 1 h incubation time, the supernatants were removed and the cells were washed carefully with ice-cold PBS buffer (2 × 1 mL, pH 7.4) followed by a subsequent glycine–HCl washing step (50 mM, pH 2.8, 2 × 1 mL well^−1^). Cells were incubated with the glycine–HCl solution for 4 min at r.t. Next, corresponding fractions were transferred into counting tubes. Subsequently, the cells were lysed by adding NaOH (2 × 1 mL well^−1^, 0.1 M) and the lysed fractions were also transferred into counting tubes. As standards, 100 µL of each radiolabeled conjugate stock solution (0.025 nmol mL^−1^, 2.5 pmol) were pipetted into counting tubes containing 2 mL PBS buffer. The activity of the standards, the surface bound fractions and the internalized fractions were measured in a Gamma counter. Means were calculated and subtracted by the background. The percentages of unspecific and specific cell-surface bound and internalized activity were calculated from the standards.

### Small-animal PET imaging

Six to eight-week old male NOD/SCID mice (17–20 g) were obtained from Charles River Laboratories (Lyon, France). Mice were provided with food and water ad libitum. LNCaP tumors were induced on the right shoulder by sub-cutaneous injection of 5 × 10^6^ cells in a 100 μL cell suspension of a 1:1 v/v mixture of media with reconstituted basement membrane (GFR BD Matrigel™, Corning BV, Amsterdam, Holland). After an average of 8–10 weeks, tumor size reached ~ 500–600 mm^3^ and the animals were used for PET imaging studies.

For PET imaging studies, mice were injected with 100 µL sterile saline formulations of 250–500 pmol [^68^Ga]Ga-**9** (1–3 MBq) by intravenous tail-vein injection and anesthetized with isoflurane (2–4% in air). PET Imaging was performed on an Albira PET/SPECT/CT scanner (Bruker, Germany) at 1 h p.i. Data were acquired in list mode. Reconstruction was performed using unweighted OSEM2D. Image analysis was performed using PMOD (V6.3.4, Bruker). Image counts per second per voxel (cps voxel^−1^) were calibrated to activity concentrations (Bq mL^−1^) by measuring a 3.5 cm cylinder phantom filled with a known concentration of radioactivity. Regions of interest (ROI) were drawn manually for the tumors, liver, heart, salivary glands, and the kidneys to measure the activity per mL for each organ.

Competitive inhibition (blocking) studies were also performed in vivo and measured using static PET imaging to investigate the specificity of the radiotracers for PSMA. As a blocking agent 2-PMPA (1 µmol mouse^−1^) was co-injected with the radiotracers (*n* = 3).

### Clinical PET/CT Imaging

PET/CT was performed approximately 1, 2, and 3 h after intravenous injection of 107 MBq of [^68^Ga]Ga-**9** and 1 h after injection of 113 MBq [^68^Ga]Ga-PSMA-11 in a 60-year-old male patient with metastatic prostate cancer. Whole body imaging extending from vertex to mid-femur was performed in 3D-ToF mode on a Biograph mCT 40 scanner (Siemens Medical Solutions, Knoxville, TN, USA) with an extended field of view of 21.4 cm. PET acquisition time was 3 min bed position^−1^. Low-dose CT acquisition was performed for attenuation correction and anatomical localization using an X-ray tube voltage of 120 kV and a modulation of the tube current (maximal tube current: 30 mA; CARE Dose 4D software Siemens Healthineers, Erlangen, Germany) followed by reconstruction with a soft tissue reconstruction kernel (Bf37) to a slice thickness of 5 mm (increment 2–4 mm). PET emission data were additionally corrected for decay, randoms and scatter and reconstructed applying an iterative 3D ordered subset expectation maximization algorithm (3 iterations; 21 subsets) withGaussian filtering to transaxial resolution of 5 mm at full width at half maximum. The matrix size was 200 × 200 mm and the reconstructed pixel size was 5.0 mm. There were no adverse events or changes in vital signs after application.

## Supplementary Information

Additional file1

## Data Availability

The datasets generated during and/or analyzed during the current study are available as Supplementary Material. Further data can be obtained from the corresponding author on reasonable request.

## Abbreviations

2-PMPA: 2-(Phosphonomethyl)pentanedioeic acid
CAF: Cancer associated fibroblasts
cGMP: Current good manufacturing practice
DIPEA: *N*,*N*-Diisopropylethylamine
FAP: Fibroblast activation protein
FAPI: Fibroblast activation protein inhibitor
HATU: O-(7-Azabenzotriazol-1-yl)-*N*,*N*,*N′*,*N′*-tetramethyluronium-hexafluorphosphate
HBED-CC: *N,N*′-Bis-[2-hydroxy-5-(carboxyethyl)benzyl]ethylenediamine-*N,N′-*diacetic acid
IC_50_: Half maximum inhibitory constant
MIP: Maximum intensity projection
NOTI: 1,4,7-Triazacyclonoane-1,4,7-tri-methylimidazole
% IA g^−^^1^: Percent injected activity per gram
p.i.: Post-injection
PSMA: Prostate specific membrane antigen
RCP: Radiochemical purity
RCY: Radiochemical yield
SAR: Sarcophagine
SUV: Standardized uptake value
TAC: Time activity curve
TFA: Trifluoroacetic acid
TIS: Triisopropylsilane
TVA: Trivaleric acid
