# Supplementary material for: Development of a homotrimeric PSMA radioligand based on the NOTI chelating platform
Source: EJNMMI Radiopharm Chem. 2024 Dec 11;9:84. doi: 10.1186/s41181-024-00314-7 (PMC11635053; doi:10.1186/s41181-024-00314-7)
Supplement: Supplementary file 1 — Additional file1 [file 41181_2024_314_MOESM1_ESM.pdf]

Supporting Information

Development of a homotrimeric PSMA radioligand based on the NOTI chelating platform

Sebastian Martin[1], Moritz-Valentin Schreck[2], Tobias Stemler[2], Stephan Maus[2], Florian Rosar[2], Caroline Burgard[2], Andrea Schaefer-Schuler[2], Samer Ezziddin[2] and Mark D. Bartholomä[2]*

[1] Department of Nuclear Medicine and Molecular Imaging, Lausanne University Hospital, Rue de Bugnon 25A, CH-1011 Lausanne, Switzerland.

[2] Department of Nuclear Medicine, Saarland University – Medical Center, Kirrbergerstrasse, D-66421, Homburg, Germany

*** Corresponding Author:**
Dr. Mark D. Bartholomä
Tel: +49-(6841)-16-24592
Fax: +49-(6841)-16-24692
E-mail: mark.bartholomae@uks.eu
Orchid-Id: 0000-0002-0361-2833

Table of Contents

HPLC chromatogramms 4

Figure S1. Representative UV/vis RP-HPLC chromatogram of **1**. 4

Figure S2. Representative UV/vis RP-HPLC chromatogram of **2**. 4

Figure S3. Representative UV/vis RP-HPLC chromatogram of **3**. 5

Figure S4. Representative UV/vis RP-HPLC chromatogram of **4**. 5

Figure S5. Representative UV/vis RP-HPLC chromatogram of **7**. 6

Figure S6. Representative UV/vis RP-HPLC chromatogram of **8**. 6

Figure S7. Representative UV/vis RP-HPLC chromatogram of **9**. 7

Figure S8. Representative radio-RP-HPLC chromatogram of [^{68}Ga]Ga-**7**. 7

Figure S9. Representative radio-RP-HPLC chromatogram of [^{68}Ga]Ga-**8**. 8

Figure S10. Representative radio-RP-HPLC chromatogram of [^{68}Ga]Ga-**9**. 8

Figure S11. Representative UV/vis RP-HPLC chromatogram of ^{nat}Ga-**7** 9

Figure S12. Representative UV/vis RP-HPLC chromatogram of ^{nat}Ga-**8** 9

Figure S13. Representative UV/vis RP-HPLC chromatogram of ^{nat}Ga-**9** 10

NMR data 11

Figure S14. ^{1}H-NMR of compound **1** in $CDCl_3$ 11

Figure S15. ^{1}H-NMR of compound **2** in d_4-MeOD. 11

Figure S16. ^{1}H-NMR of compound **3** in d_4-MeOD. 12

Figure S17. ^{1}H-NMR of compound **4** in D_2O. 12

Mass spectrometry data 13

Figure S18. HR-ESI-MS of compound **1**. 13

Figure S19. HR-ESI-MS of compound **2**. 13

Figure S20. HR-ESI-MS of compound **3**. 14

Figure S21. HR-ESI-MS of compound **7**. 14

Figure S22. LR-ESI-MS of compound **7**. 15

Figure S23. HR-ESI-MS of compound **8**. 15

Figure S24. LR-ESI-MS of compound **8**. 15

Figure S25. HR-ESI-MS of compound **9**. 16

Figure S26. LR-ESI-MS of compound **9**. 16

Figure S27. HR-ESI-MS of ^{nat}Ga-**7**. 17

Figure S28. HR-ESI-MS of ^{nat}Ga-**8**. 17

Figure S29. HR-ESI-MS of ^{nat}Ga-**9**. 18

HPLC chromatogramms

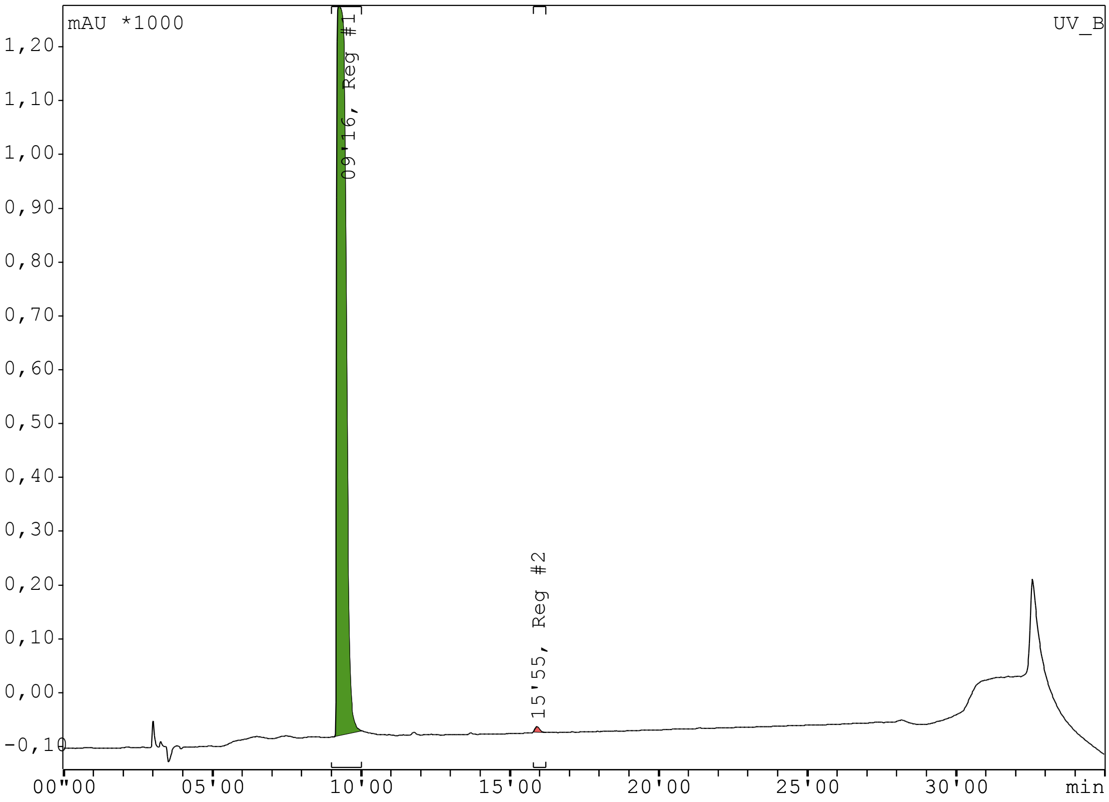

Figure S1. Representative UV/vis RP-HPLC chromatogram of **1**.

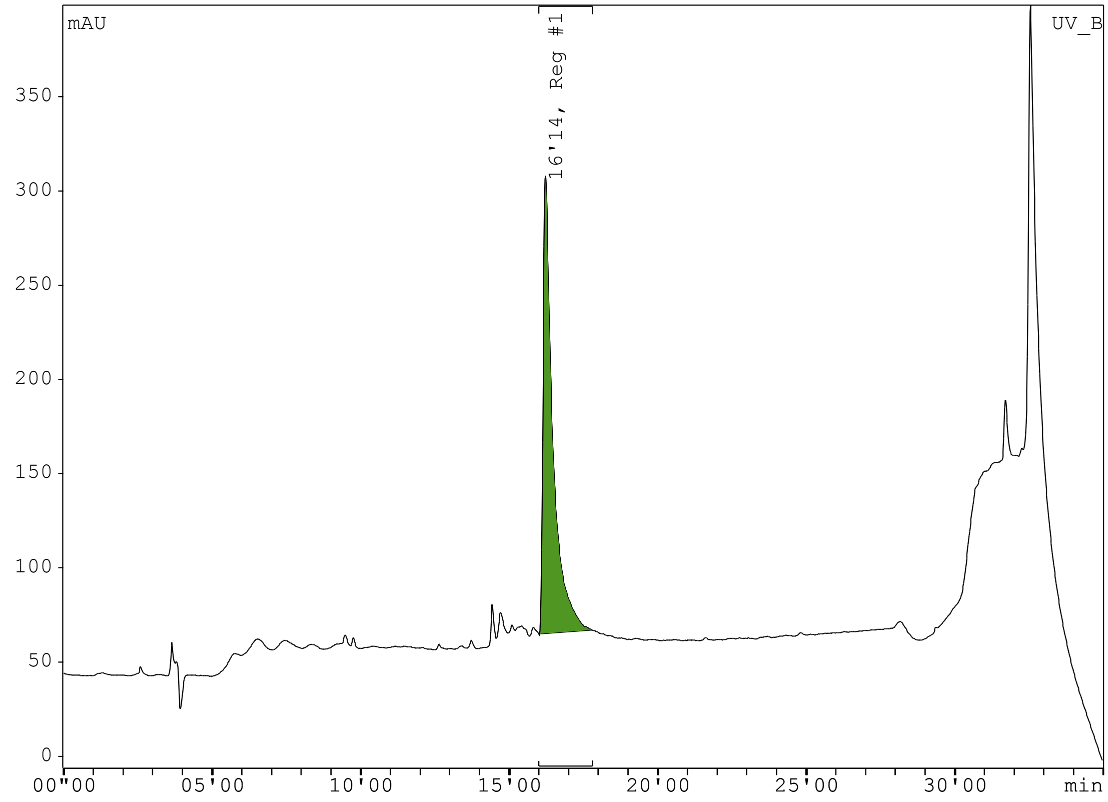

Figure S2. Representative UV/vis RP-HPLC chromatogram of **2**.

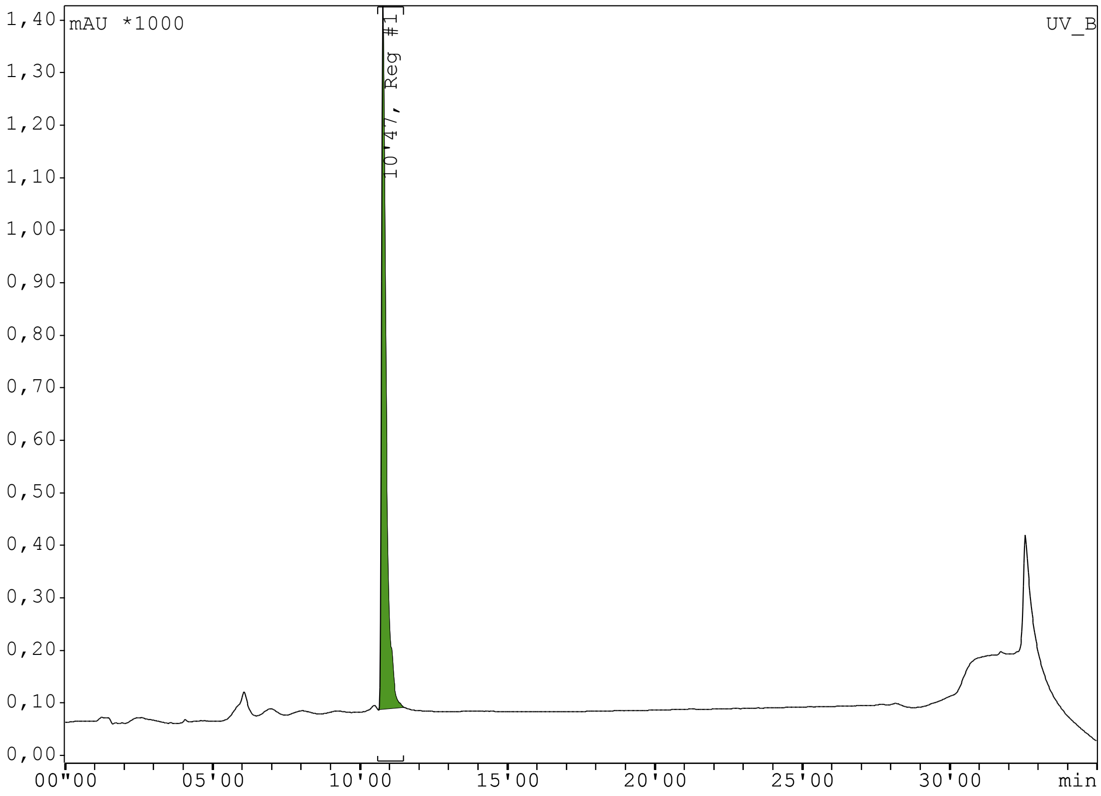

Figure S3. Representative UV/vis RP-HPLC chromatogram of **3**.

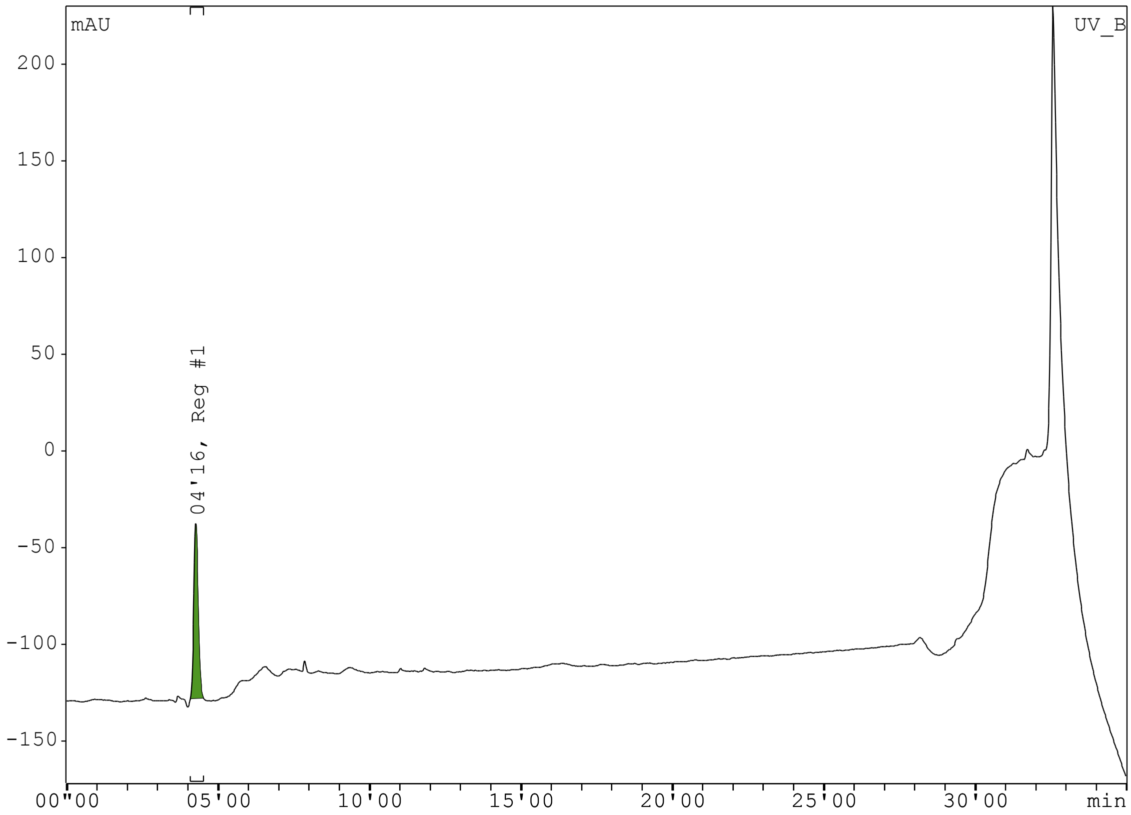

Figure S4. Representative UV/vis RP-HPLC chromatogram of **4**.

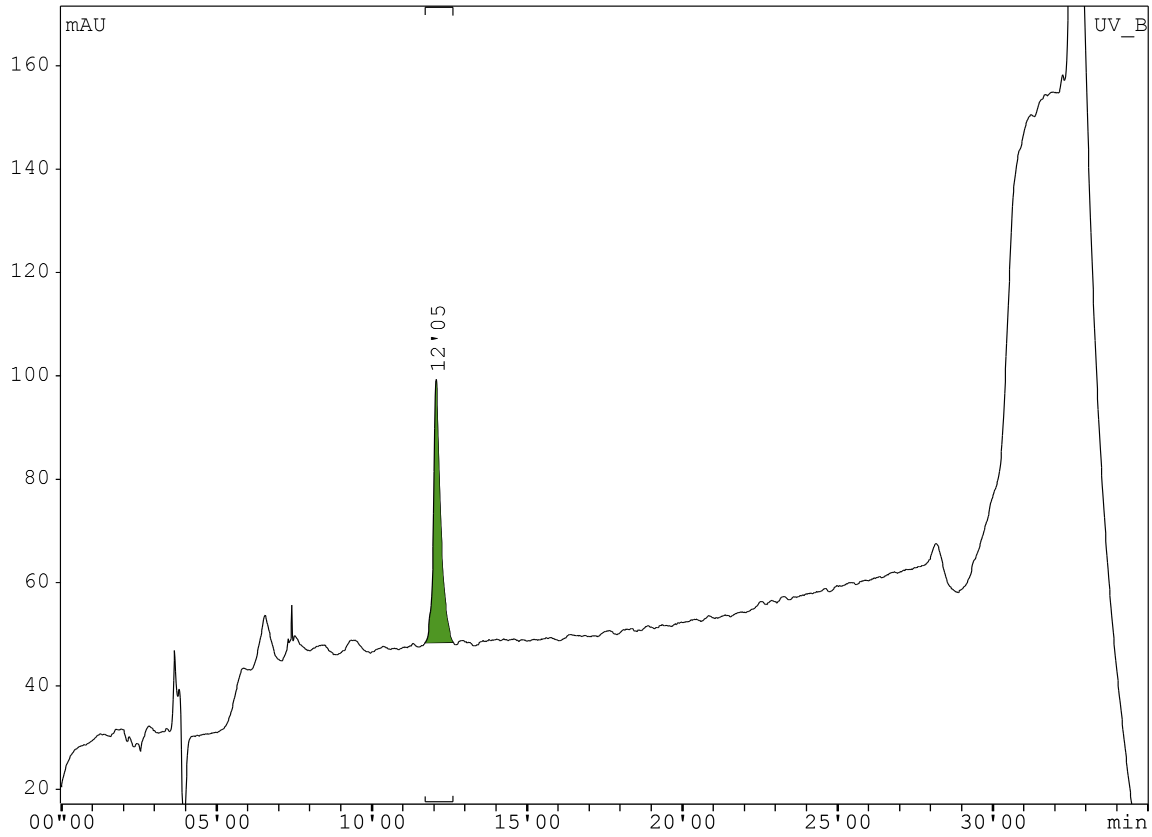

Figure S5. Representative UV/vis RP-HPLC chromatogram of **7**.

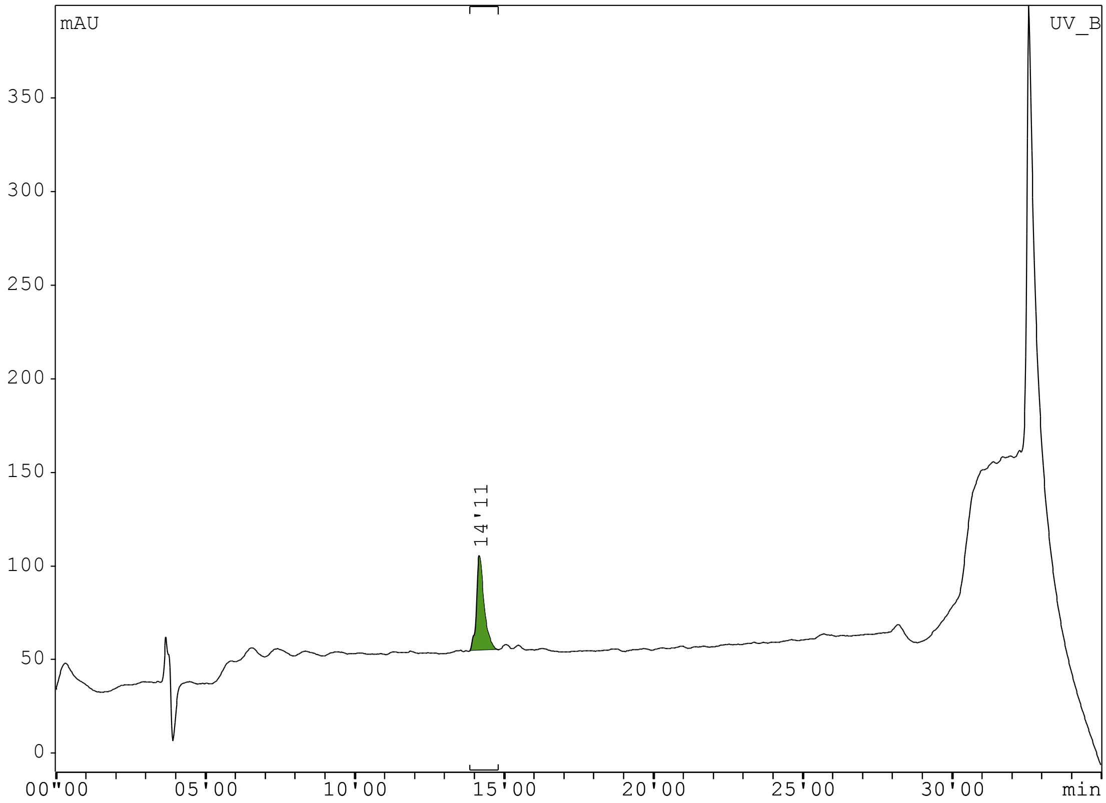

Figure S6. Representative UV/vis RP-HPLC chromatogram of **8**.

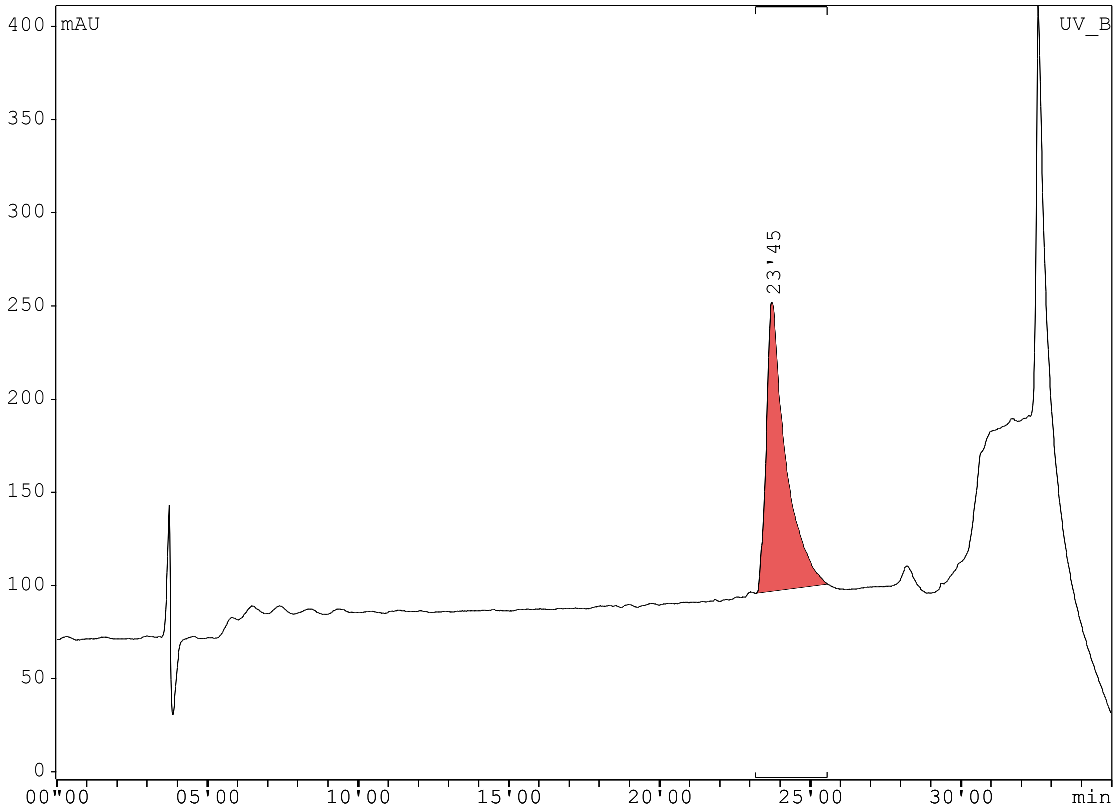

Figure S7. Representative UV/vis RP-HPLC chromatogram of **9**.

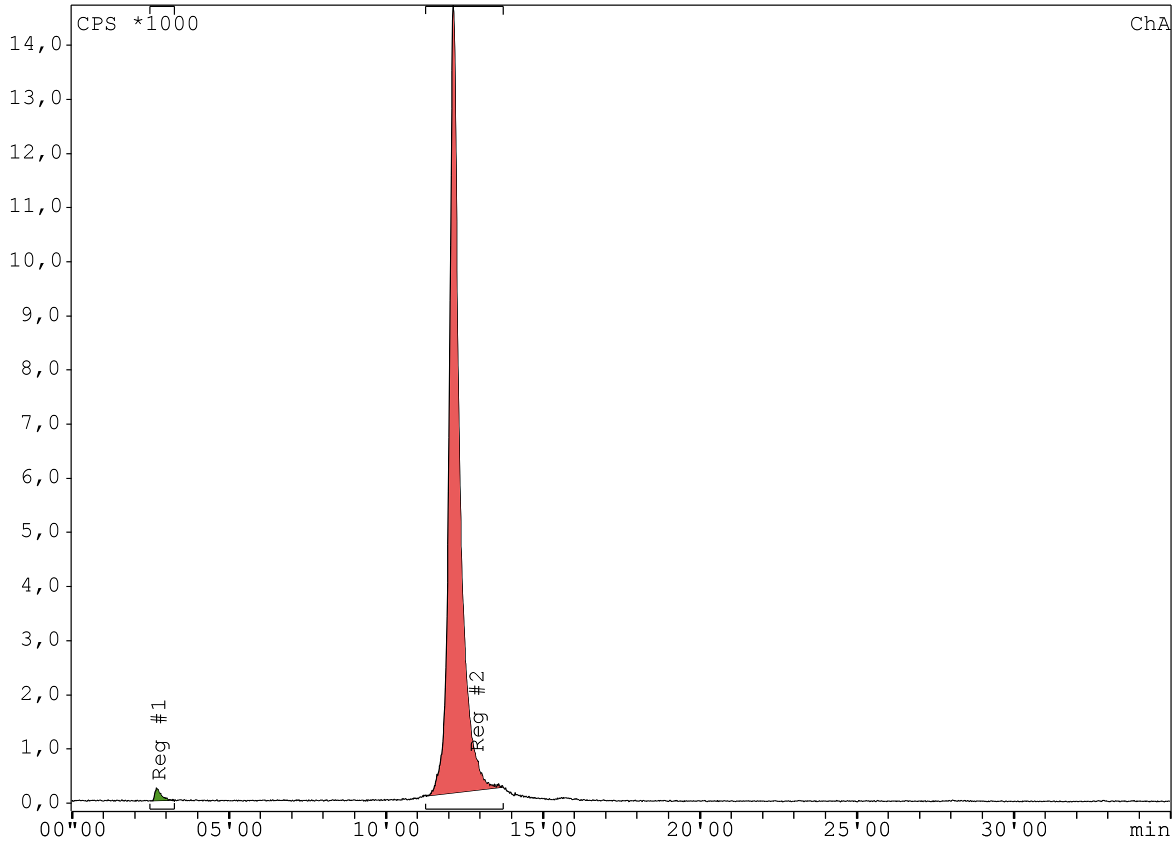

Figure S8. Representative radio-RP-HPLC chromatogram of [^{68}Ga]Ga-**7**.

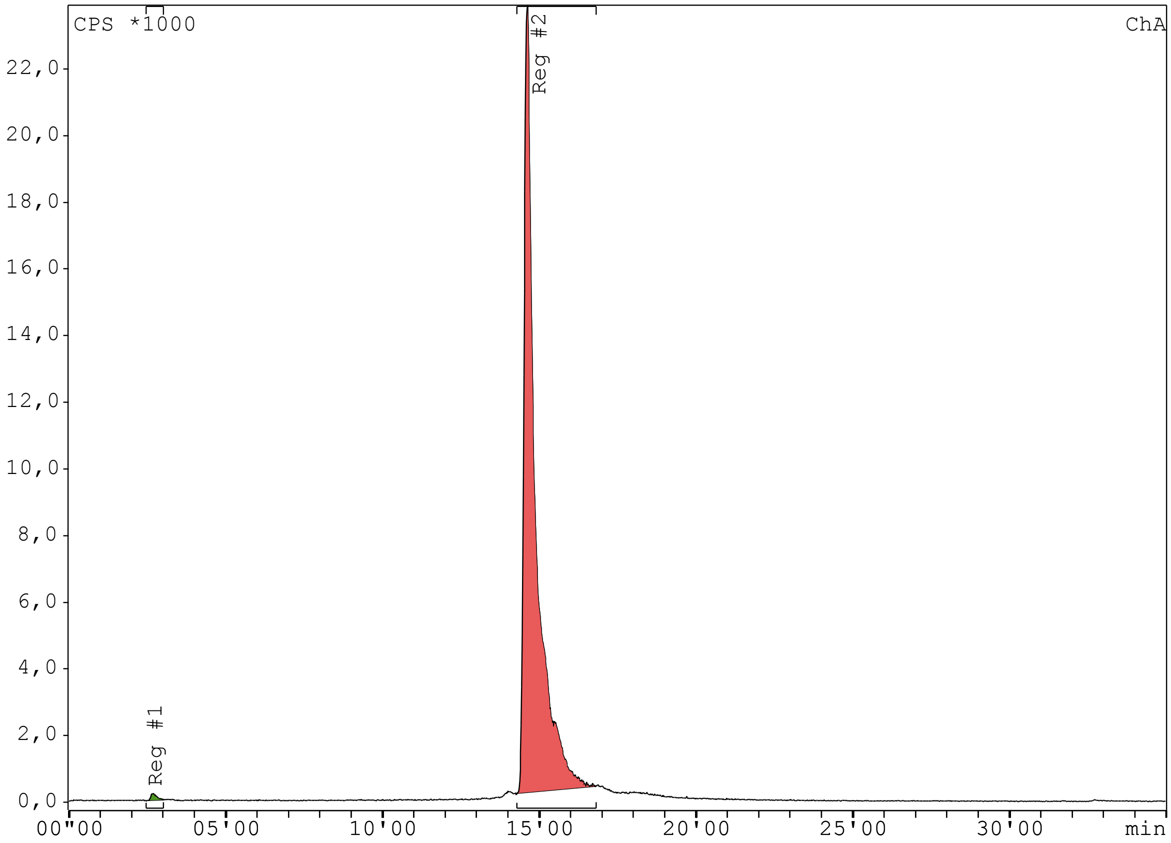

Figure S9. Representative radio-RP-HPLC chromatogram of [^{68}Ga]Ga-**8**.

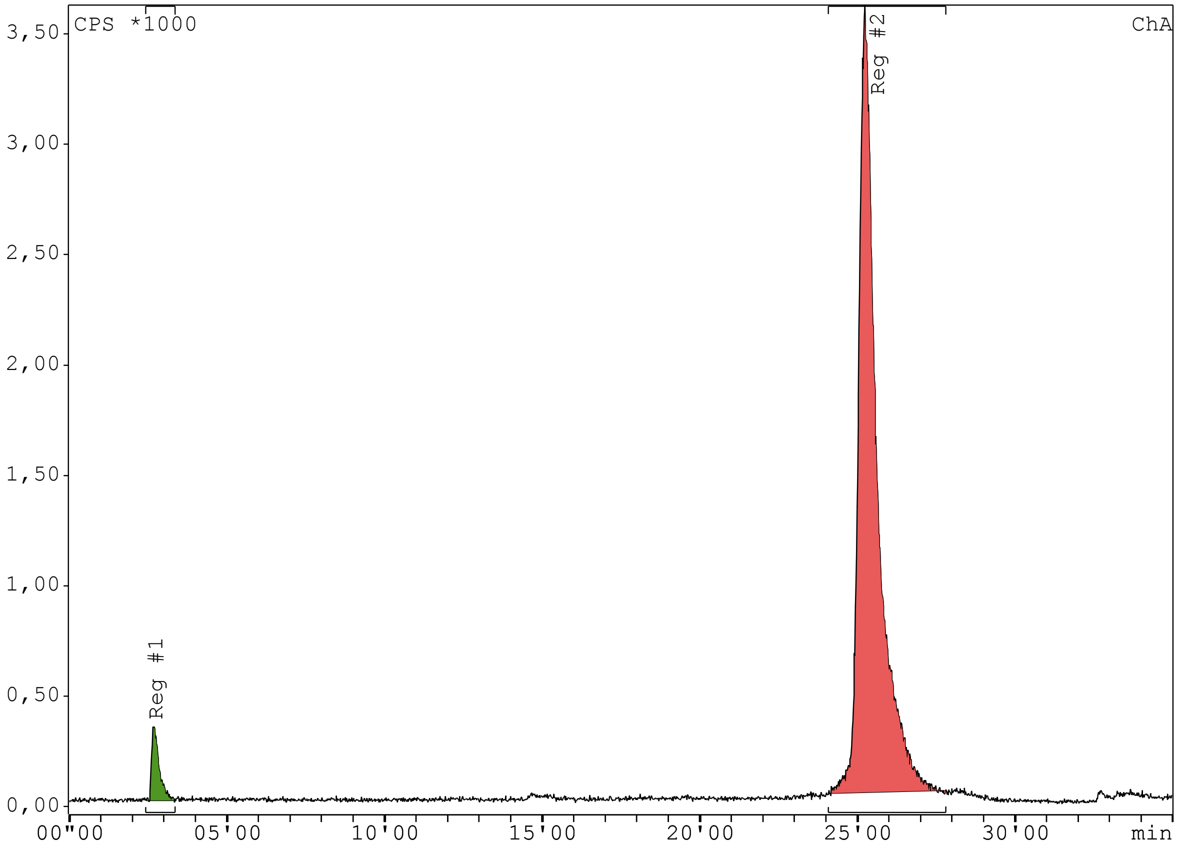

Figure S10. Representative radio-RP-HPLC chromatogram of [^{68}Ga]Ga-**9**.

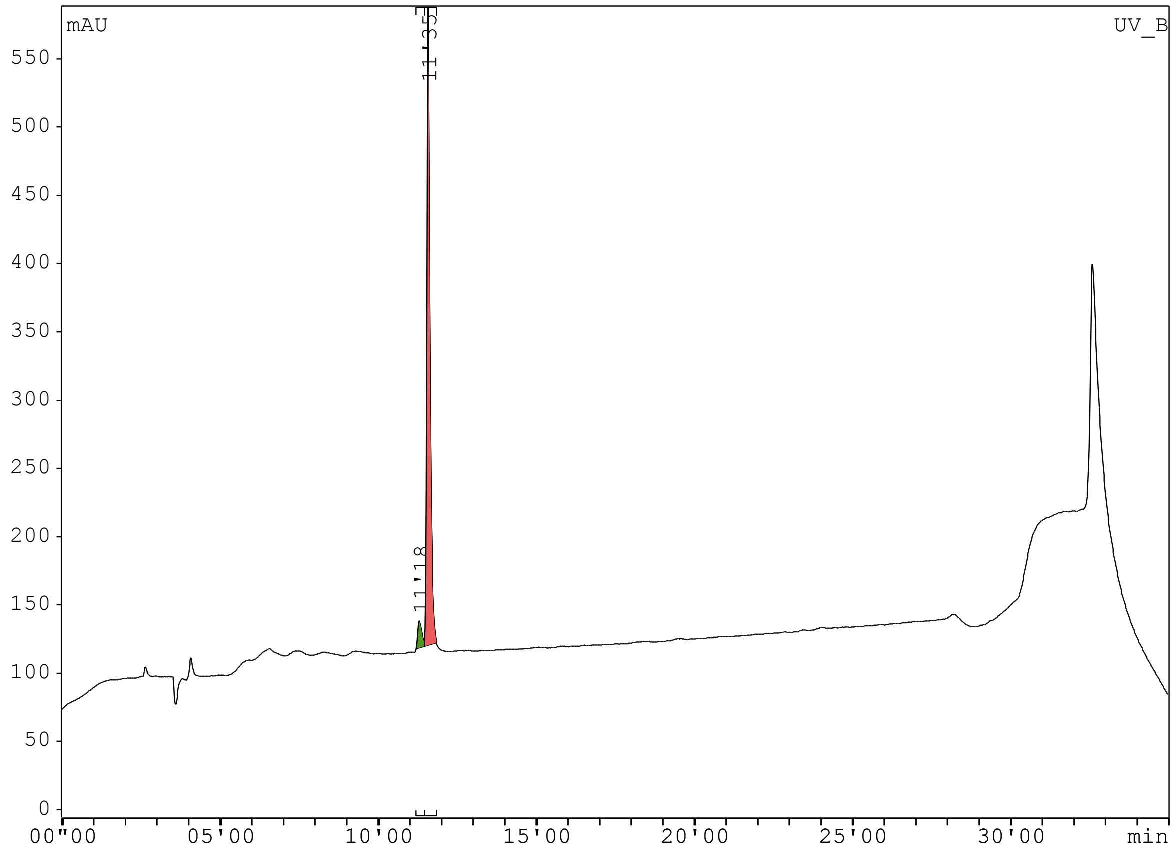

Figure S11. Representative UV/vis RP-HPLC chromatogram of ^{nat}Ga-**7**.

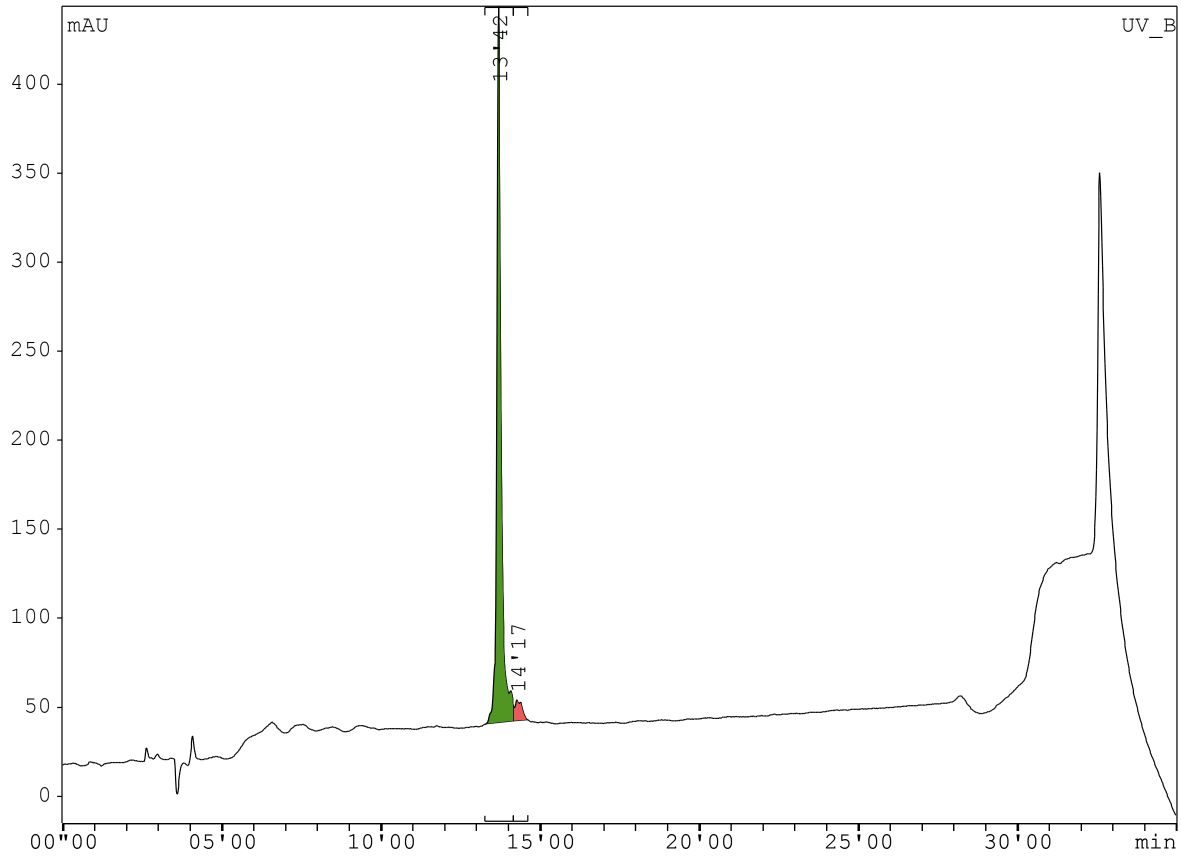

Figure S12. Representative UV/vis RP-HPLC chromatogram of ^{nat}Ga-**8**.

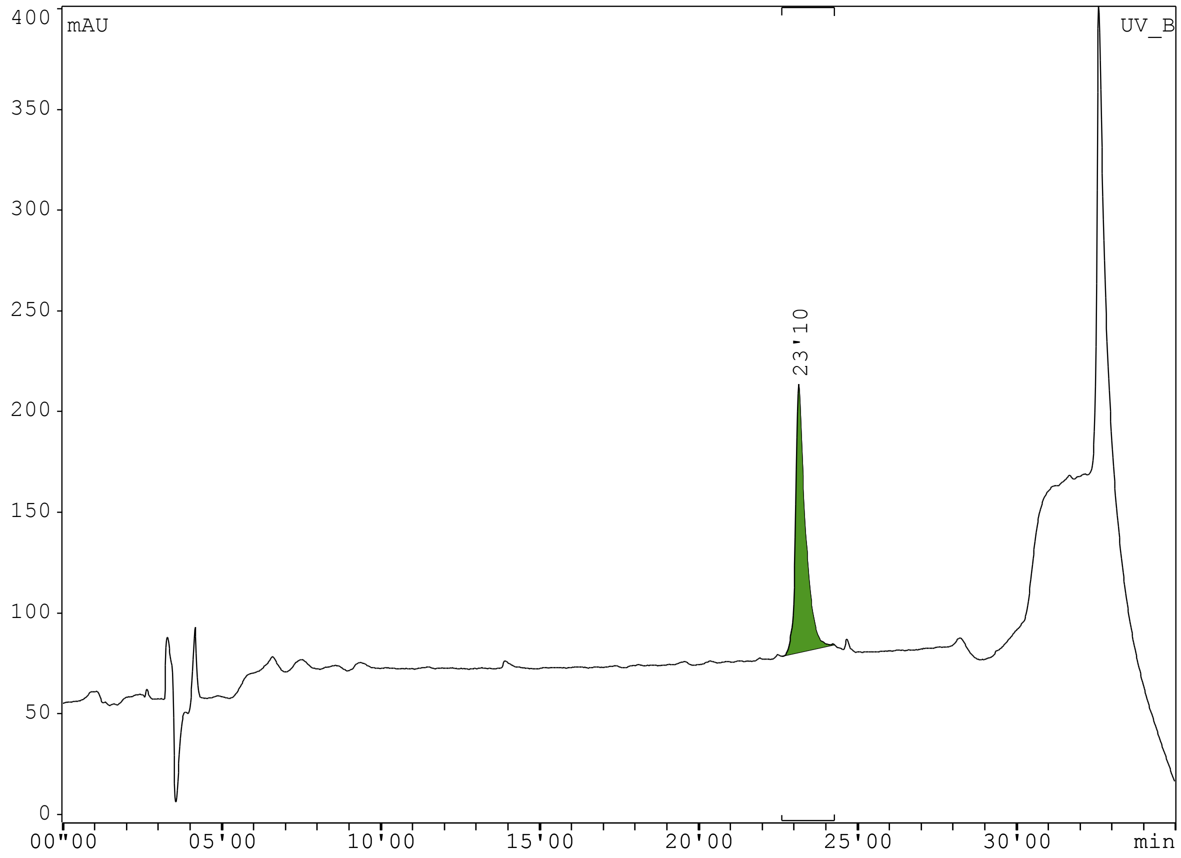

Figure S13. Representative UV/vis RP-HPLC chromatogram of ^{nat}Ga-**9**.

NMR data

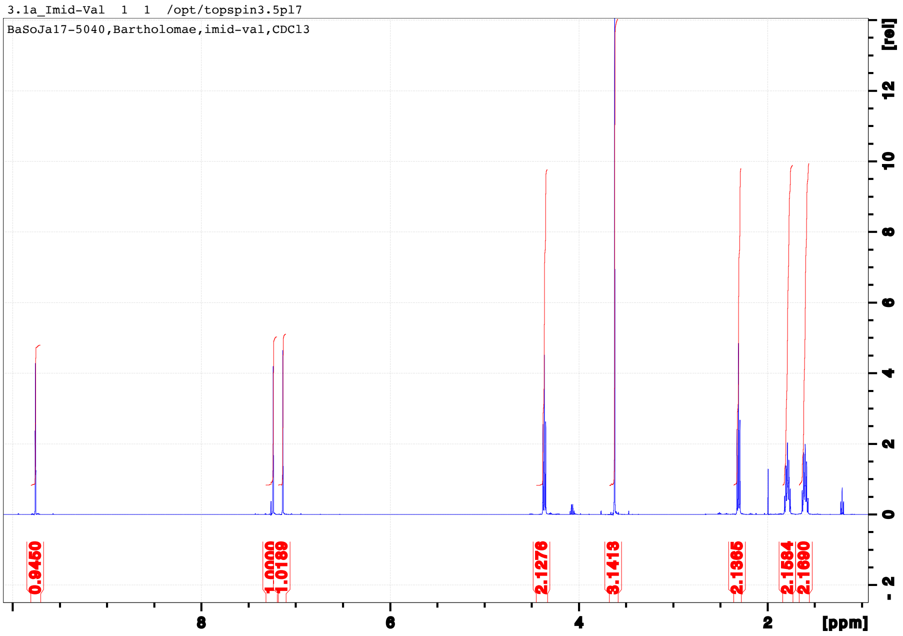

Figure S14. ^{1}H-NMR of compound **1** in $CDCl_3$.

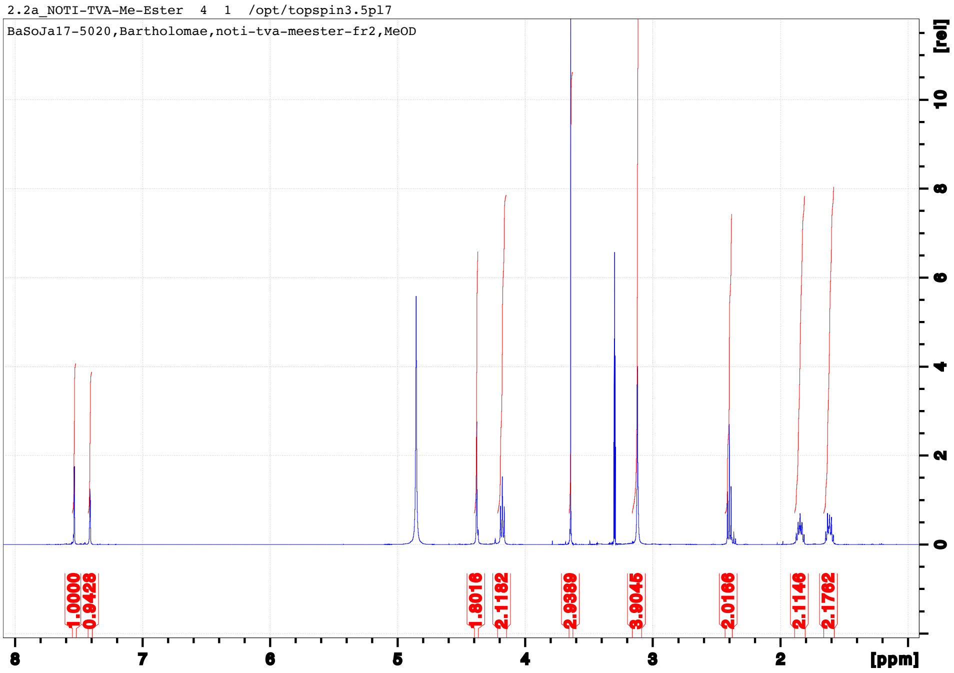

Figure S15. ^{1}H-NMR of compound **2** in d_4-MeOD.

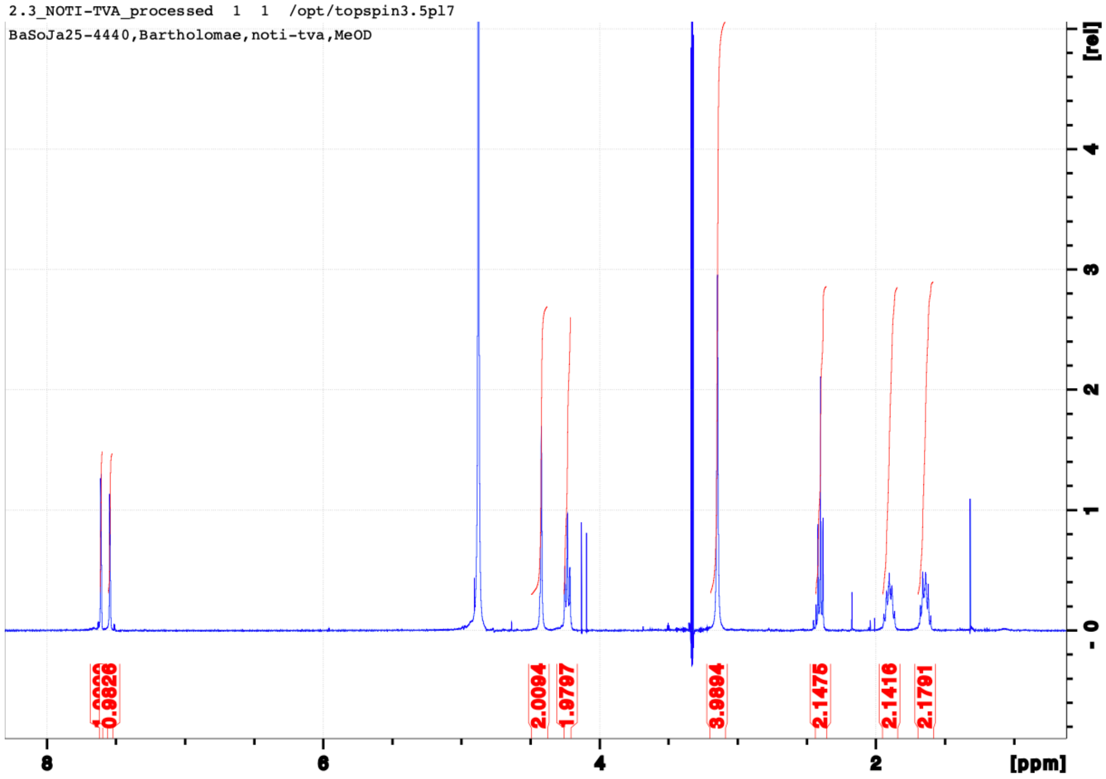

Figure S16. ^{1}H-NMR of compound **3** in d_4-MeOD.

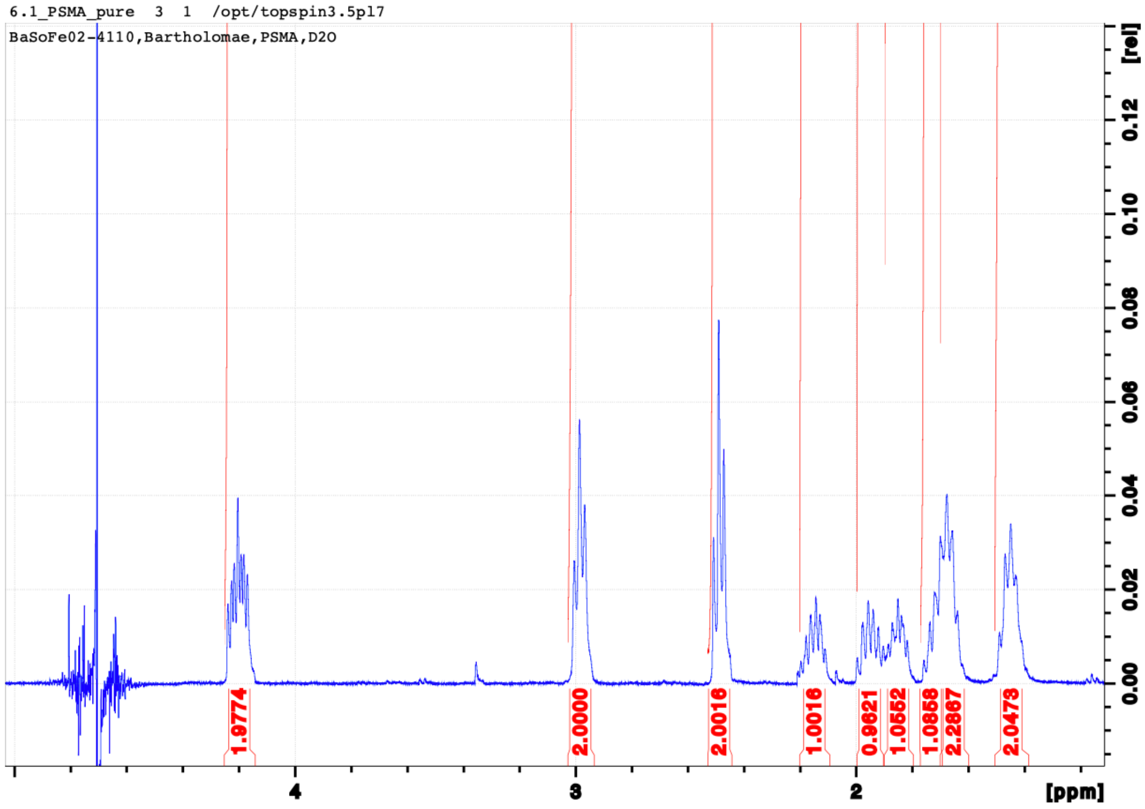

Figure S17. ^{1}H-NMR of compound **4** in D_2O.

Mass spectrometry data

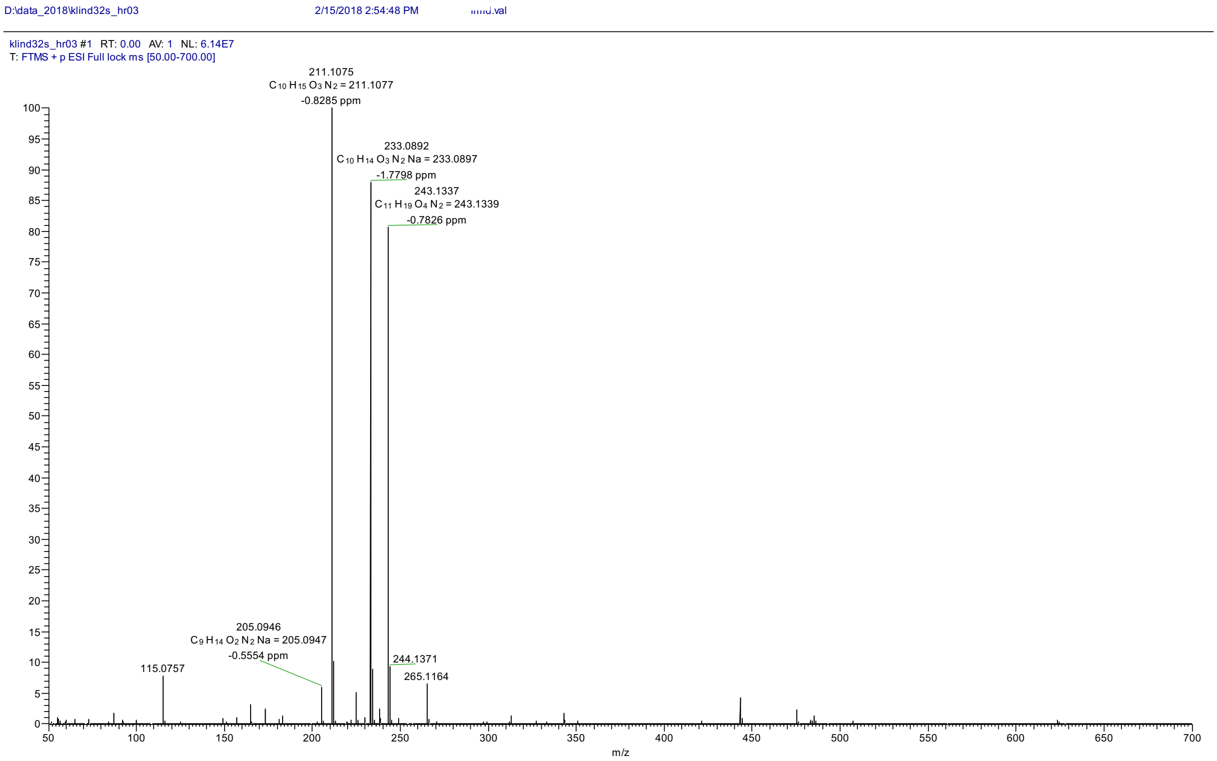

Figure S18. HR-ESI-MS of compound **1**.

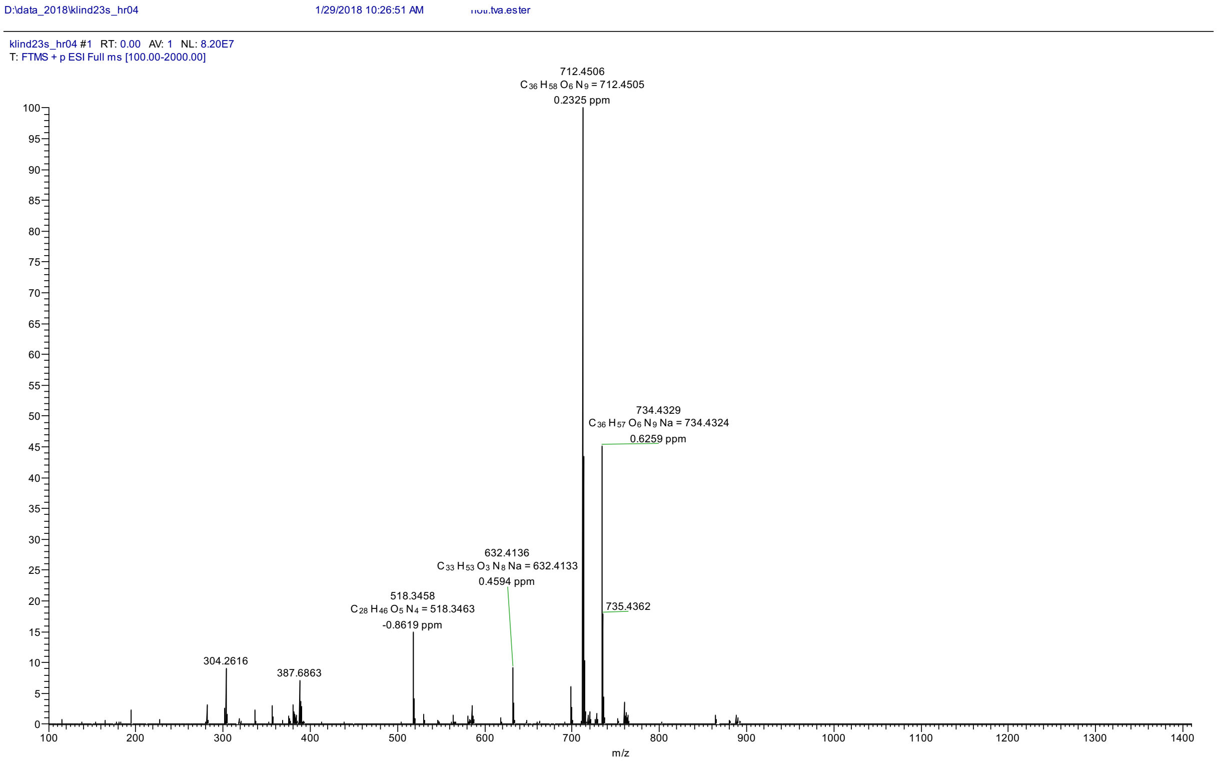

Figure S19. HR-ESI-MS of compound **2**.

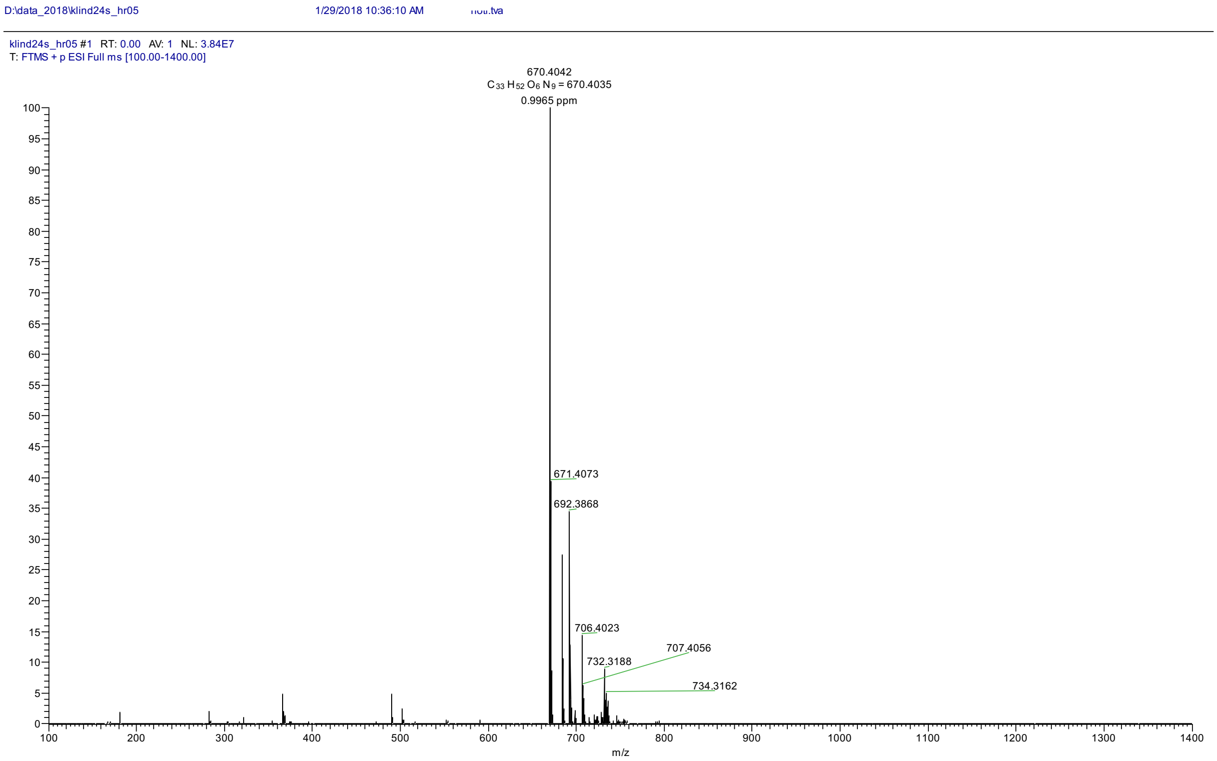

Figure S20. HR-ESI-MS of compound **3**.

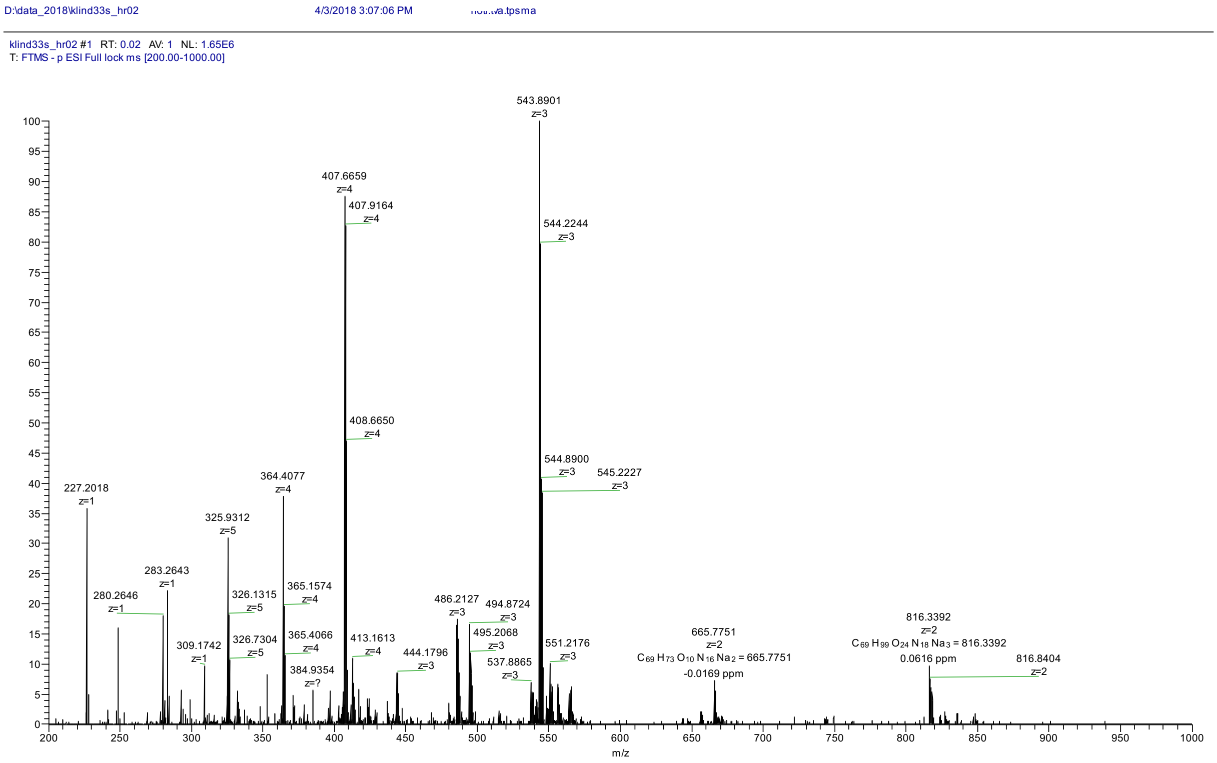

Figure S21. HR-ESI-MS of compound **7**.

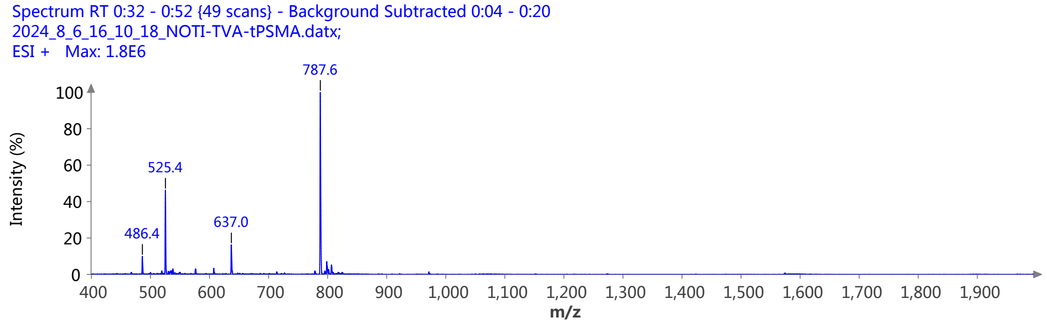

Figure S22. LR-ESI-MS of compound **7**.

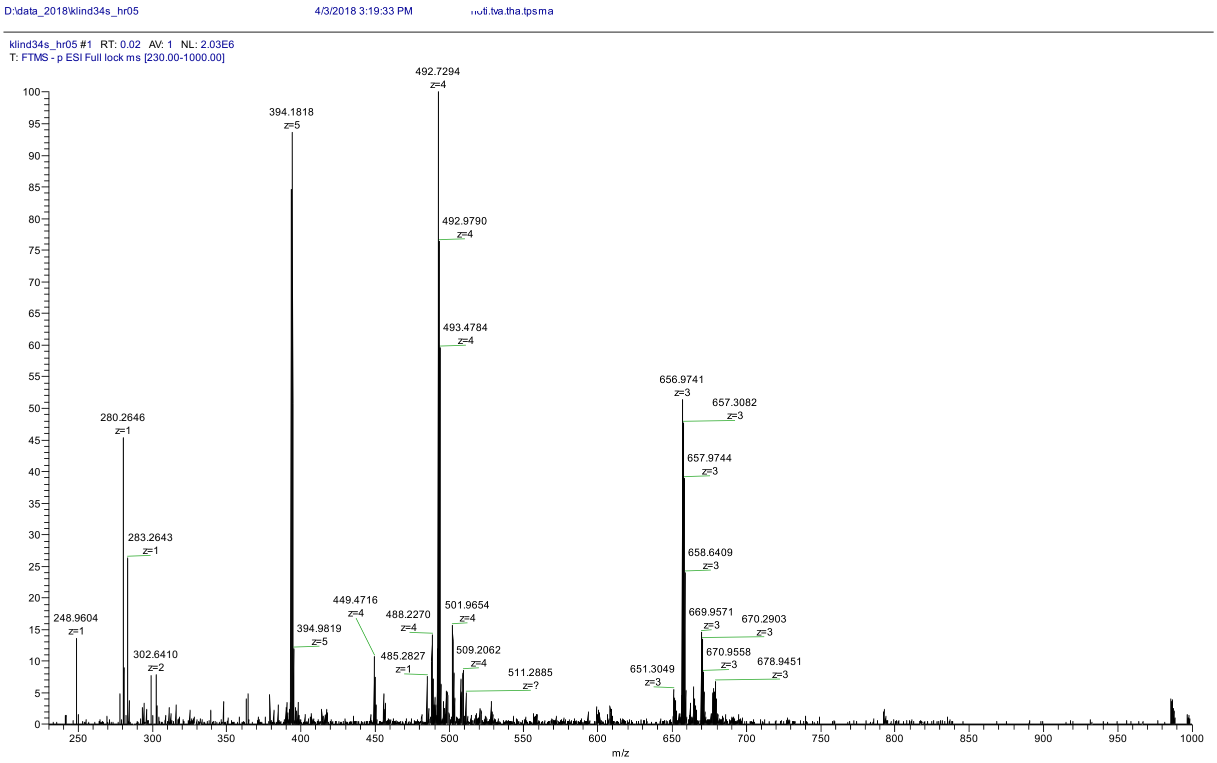

Figure S23. HR-ESI-MS of compound **8**.

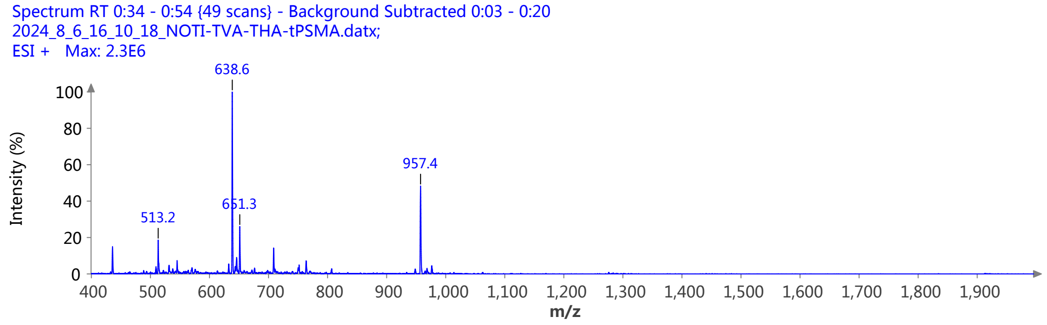

Figure S24. LR-ESI-MS of compound **8**.

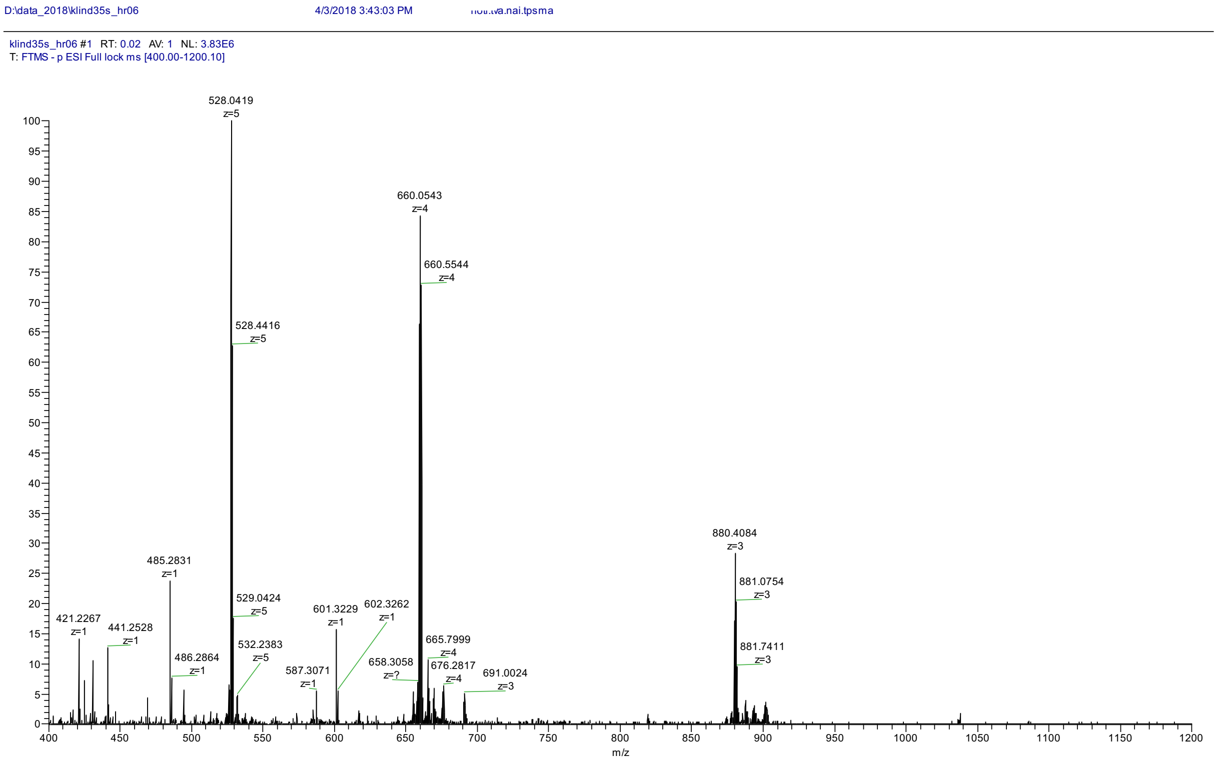

Figure S25. HR-ESI-MS of compound **9**.

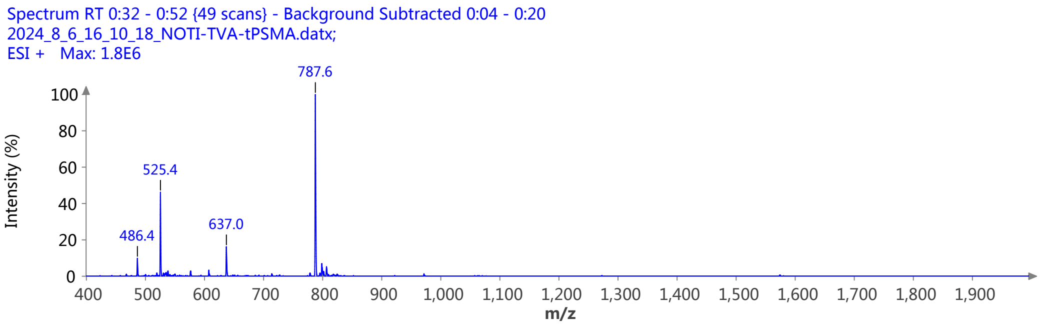

Figure S26. LR-ESI-MS of compound **9**.

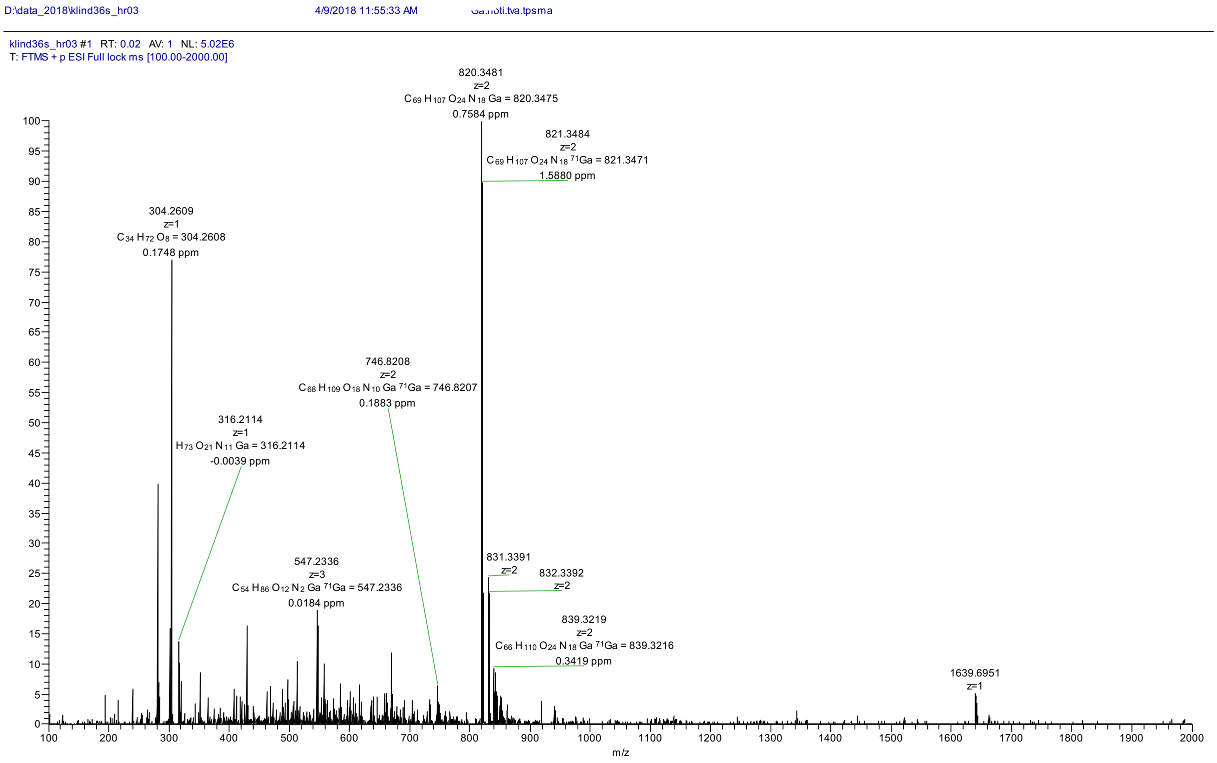

Figure S27. HR-ESI-MS of ^{nat}Ga-**7**.

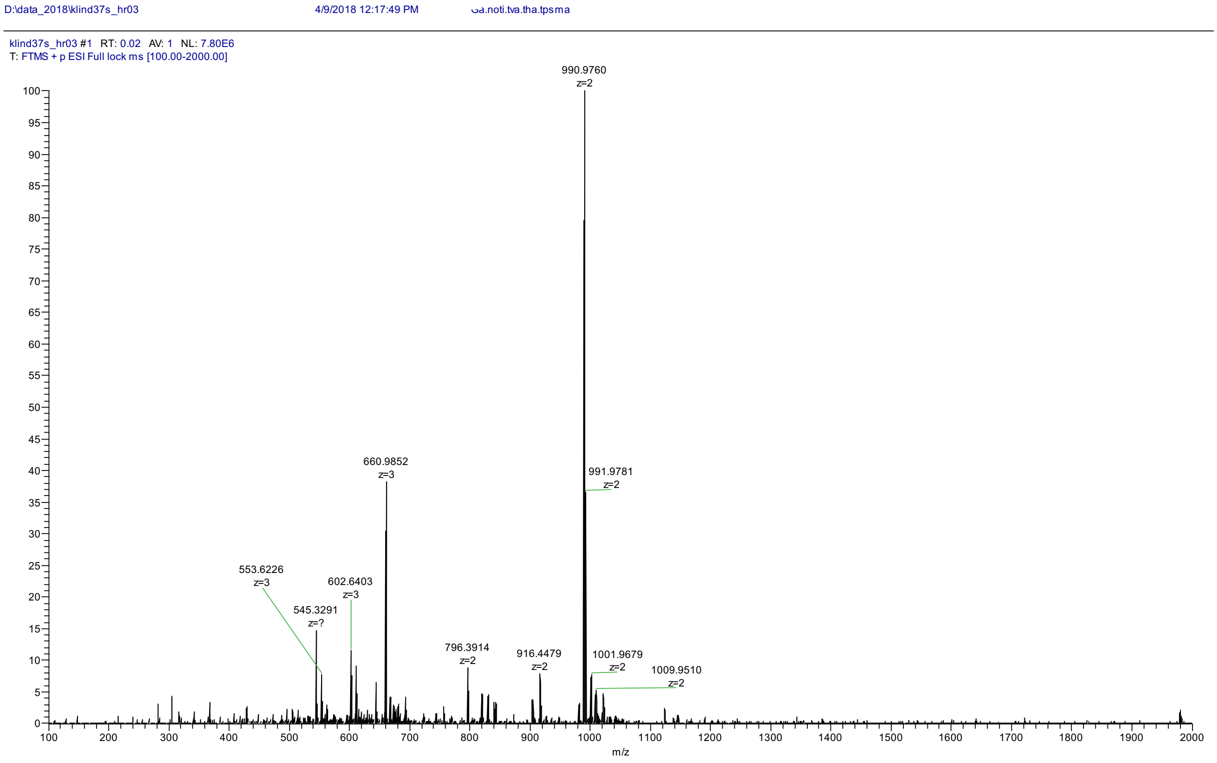

Figure S28. HR-ESI-MS of ^{nat}Ga-**8**.

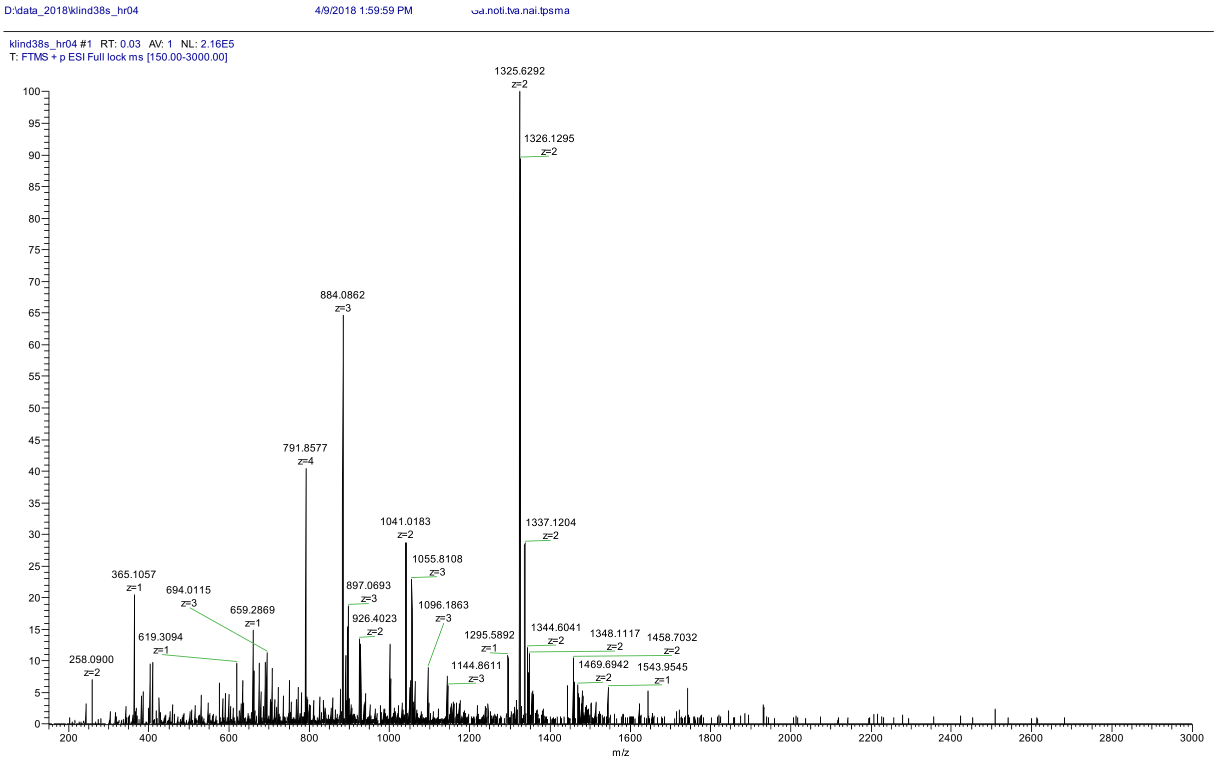

Figure S29. HR-ESI-MS of ^{nat}Ga-**9**.
